# Replication properties of a contemporary Zika virus from West Africa

**DOI:** 10.1371/journal.pntd.0012066

**Published:** 2024-07-05

**Authors:** Dana Machmouchi, Marie-Pierre Courageot, Chaker El-Kalamouni, Alain Kohl, Philippe Desprès

**Affiliations:** 1 Processus Infectieux en Milieu Insulaire Tropical (PIMIT), Université de La Réunion, INSERM U1187, CNRS 9192, IRD 249, Plateforme Technologique CYROI, Sainte-Clotilde, La Réunion, France; 2 UR7506-BioSpect, Université de Reims Champagne-Ardennes, 51100 Reims, France; 3 Centre for Neglected Tropical Diseases, Departments of Tropical Disease Biology and Vector Biology, Liverpool School of Tropical Medicine, Liverpool, United Kingdom; 4 MRC-University of Glasgow Centre for Virus Research, Glasgow, United Kingdom; University of Wisconsin-Madison, UNITED STATES OF AMERICA

## Abstract

Zika virus (ZIKV) has become a global health problem over the past decade due to the extension of the geographic distribution of the Asian/American genotype. Recent epidemics of Asian/American ZIKV have been associated with developmental disorders in humans. There is mounting evidence that African ZIKV may be associated with increased fetal pathogenicity necessitating to pay a greater attention towards currently circulating viral strains in sub-Saharan Africa. Here, we generated an infectious molecular clone GUINEA-18 of a recently transmitted human ZIKV isolate from West Africa, ZIKV-15555. The available infectious molecular clone MR766^MC^ of historical African ZIKV strain MR766-NIID was used for a molecular clone-based comparative study. Viral clones GUINEA-18 and MR766^MC^ were compared for their ability to replicate in VeroE6, A549 and HCM3 cell lines. There was a lower replication rate for GUINEA-18 associated with weaker cytotoxicity and reduced innate immune system activation compared with MR766^MC^. Analysis of chimeric viruses between viral clones stressed the importance of NS1 to NS4B proteins, with a particular focus of NS4B on GUINEA-18 replicative properties. ZIKV has developed strategies to prevent cytoplasmic stress granule formation which occurs in response to virus infection. GUINEA-18 was greatly efficient in inhibiting stress granule assembly in A549 cells subjected to a physiological stressor, with NS1 to NS4B proteins also being critical in this process. The impact of these GUINEA-18 proteins on viral replicative abilities and host-cell responses to viral infection raises the question of the role of nonstructural proteins in the pathogenicity of currently circulating ZIKV in sub-Saharan Africa.

## Author summary

Most studies with the objective to understand the biology of Zika virus (ZIKV) were carried out using the epidemic Asian genotype of this pathogen. It is currently being discussed whether ZIKV of African genotype may have epidemic potential associated a high risk of fetal pathogenicity. It is thus urgent to improve our knowledge on recently isolated ZIKV strains from West Africa. In this study, we used the sequence of a viral strain from an individual infected by ZIKV in Guinea in 2018 to generate an infectious molecular clone. Analysis of this viral clone highlighted the preponderant role of NS1 to NS4B proteins in virus replication and cell interactions with a particular focus on ZIKV-specific stress granule formation blocking activity. We believe that our data will improve our understanding of the biology of contemporary West Africa ZIKV, opening perspectives towards a better understanding of the pathogenicity of African viral strains.

## Introduction

Zika virus (ZIKV) infection is a widespread medically important mosquito-borne viral disease for which there are no vaccines nor curative treatments [1]. ZIKV circulates in Africa, Asia and more recently in the Americas, where an enzootic transmission cycle appears to involve primates, with the mosquitoes *Aedes aegypti* and less competent *Ae*des *albopictus* as primary vectors [1,2]. ZIKV strains are phylogenetically classified as African, Asian and now Asian/American genotypes [3]. In the past decade, there has been an unexpected expansion of the geographic distribution of ZIKV strains of Asian genotype and their rapid spread caused major epidemics in the South Pacific in 2013 and for the first time in Americas in 2015. Emergence of Asian ZIKV strains has been associated with polyradiculoneuropathy (Guillain-Barré syndrome) and unprecedented severe complications in fetuses and neonates grouped together under the umbrella term Congenital Zika Syndrome (CZS), with teratogenic effects presenting as microcephaly and other serious neurological abnormalities [1,3–5]. Although ZIKV transmission in humans classically involves a blood meal by infected female mosquitoes, sexual contact, blood transfusion and intrauterine transmission have been documented as non-vectored transmission routes [1].

ZIKV is an enveloped, positive, single-stranded RNA virus belonging to *Orthoflavivirus* genus of the *Flaviviridae* family [1,2]. The genomic RNA of about 11,000 nucleotides of length contains a single open reading frame flanked by highly structured 5’ and 3’ non-coding regions (NCRs) [1,2]. The 5’NCR which is essential for initiation of viral RNA translation, includes two stem-loop (SLs) which are required during viral replication and for genome cyclization. The 3’NCR is organized into several domains with a variable structural element followed by number of conserved SLs just downstream from the translation stop codon. The genomic RNA is translated into a large polyprotein precursor that is co- and post-translationally processed into three structural proteins, capsid (C), precursor membrane (prM/M) and envelope (E) protein followed by seven nonstructural (NS) proteins NS1, NS2A, NS2B, NS3, NS4A, NS4B and NS5 [1,2]. Structural proteins C, prM, and E are required for the formation of infectious viral particles whereas NS proteins play important roles in viral RNA replication, protein processing, and virion assembly [1,2]. The NS proteins also contribute to innate immune subversion strategies of ZIKV [6]. Orthoflavivirus NS1 glycoprotein exists as a membrane-associated homodimer in the endoplasmic reticulum (ER) [7]. The ER-resident NS1 protein associates with other NS proteins into viral replication complexes (VRCs) where genome replication takes place. A part of hydrophobic NS1 dimer is also found at the cell surface or exists as a soluble lipid-associated hexamer which is released into the extracellular environment, contributing to modulation of innate immune responses [7]. Membrane-associated NS2A is involved in RNA replication and virion assembly [8–10]. NS3 protein is an enzyme with both serine protease and NTPase/helicase activities [11]. The N-terminal region of NS3 has protease activity with NS2B as cofactor [12]. The ER resident NS4A protein is an essential component of viral replication complexes (VRCs), playing a role in virus-induced membrane reorganizations [13]. Membrane bound NS4B protein contributes to VRCs and host-immunomodulation [14]. Flavivirus NS5 protein functions as both RNA methyltransferase enzyme (MTase) and RNA-dependent RNA polymerase (RdRp) [15]. In host cells, ZIKV NS5 interacts with different cellular proteins, interfering with type I interferon (IFN) signaling pathways [16].

ZIKV can infect foetuses *in utero* with adverse consequences [1,3–5]. The impact of ZIKV on pregnancy through West African populations is still poorly documented. *In-utero* exposure of rhesus macaques to 1984 West Africa ZIKV has been shown to cause adverse pregnancy [17]. Aubry et al. (2021) reported that 2015 West Africa ZIKV has teratogenic effects in a mouse model [18]. The teratogenic potential of West Africa ZIKV requires urgent public health attention, with a particular focus on recently isolated viral strains. Also, ZIKV strains of African genotype were found to display high levels of transmission by *Ae*. *albopictus*, pointing at their elevated risk of introduction in regions where this vector has established itself [19,20]. Such recently uncovered features of ZIKV of African genotype are worrying and require further investigations. The risk assessment associated to African ZIKV genotype virus implies more in-depth investigations of the virological characteristics of recently isolated viral strains circulating in sub-Saharan Africa. The contemporary West Africa ZIKV strain Faranah/18 (also called ZIKV isolate 15555 or hereafter referred as ZIKV-15555) has been obtained from an individual infected by ZIKV in Republic of Guinea in 2018. Sequencing of ZIKV-15555 genome (GenBank accession number MN025403) was achieved following inoculation of a clinical sample into mosquito-cell cultures. Infectious clone technology that enables the manipulation of orthoflavivirus is an essential tool for studying molecular determinants involved in virulence and viral propagation. Reverse genetic approaches based on the Infectious-Subgenomic-Amplicons (ISA) method have been successfully used to produce ZIKV infectious clones [21,22]. Here, ISA was used to generate an infectious molecular clone GUINEA-18 based on the ZIKV-15555 genome sequence. The historical African ZIKV strain MR766 has been isolated from a non-human primate in Uganda in 1947 [23]. Although variant virus MR766-NIID was passaged 146 times in suckling mice, the high neuropathogenic properties of MR766-NIID was not the result of mouse adaptation [23,24], and the infectious molecular clone MR766^MC^ produced in this study represents the full genome of MR766-NIID [21,22,25]. Comparative sequence analysis revealed that ZIKV strains ZIKV-15555 and MR766-NIID share 93% nucleotide similarity and 98.5% amino-acid identity. Studies on GUINEA-18 in different cell lines have shown differences in replication properties and host-cell interactions compared to MR766^MC^. We identified NS1 to NS4B proteins as playing a key role in GUINEA-18 replication strategy, pointing to key differences between African isolates.

## Materials and methods

### Cells and antibodies

Human embryonic kidney HEK-293T (ATCC, CRL-1573), human carcinoma epithelial lung A549 (Invivogen Inc, Toulouse, France), human microglial clone 3 HCM3 (ATCC, CCL-3304) and monkey kidney VeroE6 (CCL-81, ATCC, Manassas, VA, USA) cells were grown in Dulbecco’s modified Eagle’s medium (DMEM) growth medium supplemented with 10% (HEK-293T, A549 and HCM3 cells) or 5% (VeroE6 cells) of heat-inactivated fetal bovine serum (FBS, Dutscher, Brumath, France), and antibiotics (PAN Biotech Dutscher, Brumath, France) at 37°C under a 5% CO_2_ atmosphere. HEK-293T, A549 and HCM3 cells were a generous gift of Dr N. Jouvenet (Institut Pasteur, Paris). The mouse anti-*pan* orthoflavivirus envelope E protein monoclonal antibody (mAb) 4G2 was produced by RD Biotech (Besançon, France). The mouse anti-FLAG antibody was purchased from Abcam (Cambridge, UK). Donkey IgG anti-mouse IgG Alexa Fluor 488 or Alexa Fluor 594 and donkey IgG anti-rabbit IgG Alexa Fluor 594 secondary antibodies were purchased from Invitrogen (Thermo Fisher Scientific, Illkirch-Graffenstaden, France). Anti-mouse IgG-horseradish peroxidase (HRP)-conjugated secondary antibody was purchased from Abcam (Cambridge, UK). Blue-fluorescent DNA stain 4’,6-diamidino-2-phenylindole (DAPI) was purchased from Euromedex (Souffelweyersheim, France). Lipofectamine 3000 (Thermo Fisher Scientific, Illkirch-Graffenstaden, France) was used for transfection, according to the manufacturer’s instructions.

### Design of infectious molecular clones of ZIKV

The infectious molecular clone MR766^MC^ of ZIKV strain MR766-NIID (Accession n° LC002520) was generated by reverse genetic approach using the ISA method [21]. The same strategy was used to produce an infectious molecular clone GUINEA-18 of viral strain ZIKV-15555. Because the available ZIKV-15555 genome sequence (Accession n°MN025403) is lacking for the 14 first and the 86 last nucleotides of viral genomic RNA, the ends of 5’ and 3’ NCRs of GUINEA-18 were completed with MR766^MC^ sequences. The subgenomic amplicons Z-1^GUINEA-18^, Z-23^GUINEA-18^ and Z-4^GUINEA-18^ were designed to mimic Z-1^MR766^, Z-23^MR766^ and Z-4^MR766^ amplicons that were generated to produce MR766^MC^ [21]. Information on viral protein sequences encoded by different amplicons is available in S1 Fig. The amplicon Z-1^GUINEA-18^ includes the CMV promoter immediately adjacent to the 5’NCR followed by the residues 1 to 712 of viral polyprotein. The Z-23^GUINEA-18^ codes for residues 702 to 2684 of viral polyprotein. The amplicon Z-4^GUINEA-18^ codes for residues 2674 to 3423 of viral polyprotein followed by the 3’NCR of viral genome and ended by a hepatitis delta virus ribozyme and then a SV40 poly(A) signal. The Z-1/Z-23 and Z-23/Z4 amplicons match together on at least 40 nucleotides (S1 Fig). Also, the GUINEA-18 and MR766^MC^ amplicons can match between them in order to generate chimeric viruses between the two infectious molecular clones. The different Z1, Z-23 and Z-4 amplicons were chemically synthetized and inserted into plasmid pUC57 by Genecust (Boynes, France). Plasmid sequences were verified by Sanger method.

### Recovery of infectious ZIKV from molecular clones

The Z-1^GUINEA-18^, Z-23^GUINEA-18^ and Z-4^GUINEA-18^ amplicons were amplified by PCR from their respective plasmids using a set of specific primers (S1 Table). The purified PCR products were co-transfected in HEK-293T cells using Lipofectamine 3,000 (ThermoFisher, France). After 4 days, cell supernatants were recovered and used to infected VeroE6 cells in a first round of amplification (P1). After 3 to 5 days, P1 was recovered and amplified on VeroE6 cells for another 2 to 3 days to produce a working virus stock P2. The resulting P2 virus was designed hereafter as GUINEA-2018. Virus stocks P2 grown on VeroE6 cells were used in this study. Virus stock titers in plaque forming unit per ml (PFU.mL^-1^) were determined by a standard plaque-forming assay on VeroE6 cells as previously described [26].

### Production of chimeric and mutant ZIKV

**Fig 1:**
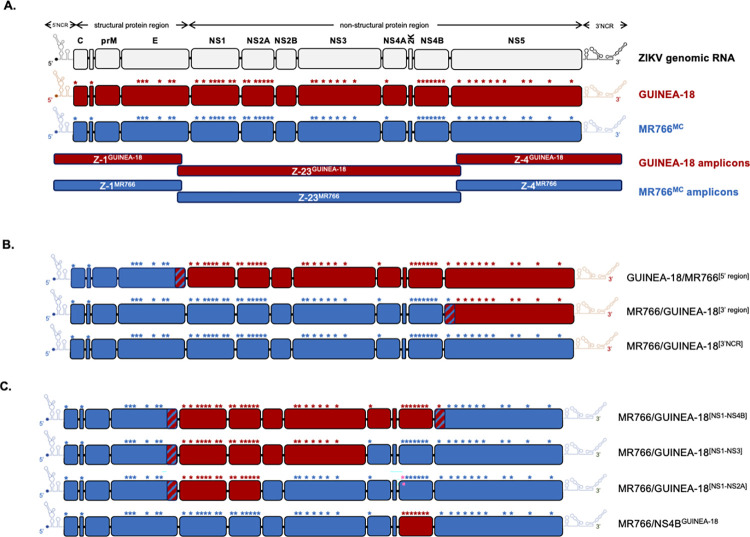
Description of infectious molecular clones GUINEA-18 and MR766^MC^. Generation of chimeric ZIKV molecular clones are shown along with infectious molecular clones GUINEA-18 and the MR766^MC^. *In* (**A**), schematic representation of ZIKV GUINEA-18 (red) and MR766^MC^ (blue) genomes. The ZIKV structural and nonstructural proteins are indicated. NCR: non-coding region. The red and blue stars show the location of the 49 amino-acid substitutions that differentiate the GUINEA-18 and MR766^MC^ polyproteins, respectively are shown. The three amplicons Z-1, Z-23, and Z-4 covering the GUINEA-18 and MR766^MC^ genomes are shown. *In* (**B**), the chimeric GUINEA-18/MR766^[5’region]^ virus contains the 5’NCR and structural protein region of MR766^MC^ inserted into GUINEA-18 backbone. The chimeric MR766/GUINEA-18^[3’region]^ virus contains the GUINEA-18 NS5 gene followed by the 3’NCR inserted into MR766^MC^ backbone. Site-directed mutagenesis was performed on 3’NCR MR766 to introduce the 3’ NCR mutations of GUINEA-18 leading to MR766/GUINEA-18^[3’NCR]^ mutant. The hatched boxes show the locations the overlapping sequences between Z-1/Z-23 and Z-23/Z-4 amplicons. *In* (**C**), the chimeric MR766/GUINEA-18^[NS1-NS4B]^, MR766/GUINEA-18^[NS1-NS3]^, MR766/GUINEA-18^[NS1-NS2A]^ and MR766/NS4B^GUINEA-18^ viruses contain different GUINEA-18 NS proteins inserted into MR766^MC^ backbone. The hatched boxes show the overlapping sequences between Z-1/Z-23 and Z-23/Z-4 amplicons. Site-directed mutagenesis was performed on Z-23 amplicons to generate chimeric MR766^MC^ viruses with GUINEA-18 NS1/NS3, NS1/NS2A or NS4B gene.

The design of chimeric and mutant ZIKV is shown in Fig 1. The Z-1^MR766^, Z-23^GUINEA-18^ and Z-4^GUINEA-18^ amplicons were used to produce a chimeric GUINEA-18 virus containing the 5’ region of MR766^MC^. The Z-1^MR766^, Z-23^MR766^ and Z-4^GUINEA-18^ amplicons were used to produce a chimeric MR766^MC^ virus containing the 3’ region of GUINEA-18. The Z-1^MR766^, Z-23^GUINEA-18^ and Z-4^MR766^ amplicons were used to produce a chimeric MR766^MC^ virus in which NS1 to NS4B proteins followed by the N-terminal region of NS5 protein were replaced by the GUINEA-18 proteins. To produce chimeric MR766^MC^ viruses containing GUINEA-18 NS1/NS2A or NS1/NS3 proteins, nested PCR was performed on Z-23^GUINEA-18^ and Z-23^MR766^ amplicons into plasmids using specific primers (S1 Table) that were designed so that the 3’ ends of Z-23^GUINEA-18^ amplicon sub-fragments coding for NS1 to NS2A or NS3 proteins match with the 5’ ends of Z-23^MR766^ amplicon sub-fragments coding for NS3 or NS2B to N-terminal part of NS5 (Fig 1). Transfection with Z-1^MR766^ amplicon, Z-23^GUINEA-18^ sub-fragment amplicon, Z-23^MR766^ sub-fragment amplicon, and Z-4^MR766^ amplicon was required for the production of chimeric MR766^MC^ viruses. To produce a mutant MR766^MC^ virus containing GUINEA-18 NS4B protein, site-directed mutagenesis was performed on Z-23^MR766^ amplicon inserted into plasmid in order to introduce the seven amino-acid substitutions of GUINEA-18 NS4B protein (S1 Table). Transfection with Z-1^MR766^ amplicon, mutant Z-23^MR766^ amplicon, and Z-4^MR766^ amplicon was carried out for the production of mutant MR766^MC^ virus with GUINEA-18 NS4B protein. To produce a mutant MR766^MC^ virus with the 3’NCR of GUINEA-18, site-directed mutagenesis was performed on the Z-4^MR766^ amplicon to introduce the eight mutations that differentiate the 3’NCRs of GUINEA-18 and MR766^MC^. Transfection with Z-1^MR766^, Z-23^MR766^, and mutant Z-4^MR766^ amplicon was then carried out for the production of mutant MR766^MC^ virus with 3’NCR of GUINEA-18. Mutagenesis and sequencing of mutant plasmids were performed by Genecust (Boynes, France).

### RT-qPCR

Total RNA was extracted from cells using RNeasy kit (Qiagen) and reverse transcription was performed using random hexamer primers (intracellular viral RNA) and MMLV reverse transcriptase (Life Technologies). Quantitative PCR was performed on a ABI7500 Real-Time PCR System (Applied Biosystems, Life Technologies, Villebon-sur-Yvette, France). Data was normalized using 36B4 gene encoding RPL0 protein as housekeeping gene. For each single-well amplification reaction, a threshold cycle (Ct) was calculated using the ABI7500 program (Applied Biosystems, Life Technologies) in the exponential phase of amplification. Relative changes in gene expression were determined using the 2∂∂*C*t method and reported relative to the control. The primers used in this study are listed in S1 Table.

### Recombinant NS4B protein

Mammalian codon-optimized genes coding for ZIKV 2K-NS4B (residues 2246 to 2527 from viral polyprotein) from viral strains MR766, ZIKV-15555, or epidemic Brazilian 2015 strain BeH819015 (Accession n° KU365778) were established using *H*. *sapiens* codon usage as reference. A glycine-serine spacer followed by a FLAG tag were inserted in-frame at the C-terminus of recombinant NS4B protein. Gene synthesis and cloning into *Nhe*-I and *Not*-I restriction sites of the pcDNA3.1-hygro (+) vector plasmid to generate recombinant plasmids pcDNA3/MR766.NS4B, pcDNA3/ZIKV-15555.NS4B, and pcDNA3/BeH819015.NS4B were performed by Genecust (Boynes, France). A deletion mutant coding for ZIKV-15555 NS4B protein without the 2K peptide (residues 2247 to 2269 from viral polyprotein) was cloned into pcDNA3.1-hygro (+) to generate pcDNA3/(Δ2K)-ZIKV-15555.NS4B. The cloning and the sequencing by Sanger method of plasmids were performed by Genecust (Boynes, France). Both A549 and HCM3 cells were transfected with plasmids by using Lipofectamine 3000.

### eGFP-G3BP fusion protein cloning

Mammalian codon-optimized gene coding for enhanced green fluorescent protein (eGFP) with the A206K monomeric mutation (Accession n° AAB02572) followed by Ras GTPase-activating protein-binding protein 1 (G3BP1) protein (Accession n° U32519) was established using *H*. *sapiens* codon usage as reference. A glycine-serine spacer was inserted in-frame between eGFP and G3BP proteins. A synthetic gene coding for fusion protein eGFP-G3BP1 was delivered by Genecust (Boynes, France). The synthetic gene was inserted into *EcoR*-I and *Not*-I restriction sites of the pcDNA3.1- vector plasmid to generate recombinant plasmid pcDNA3/eGFP-G3BP. The cloning and the sequencing by Sanger method of pcDNA3/eGFP-G3BP were performed by Genecust (Boynes, France). Endotoxin-free plasmids were delivered by Genecust (Boynes, France). Endotoxin-free plasmid was delivered by Genecust (Boynes, France). A549 cells were transfected with pcDNA3/eGFP-G3BP using Lipofectamine 3,000.

### Confocal immunofluorescence assay

Cells seeded on coverslips were fixed with 3.7% paraformaldehyde (PFA) in PBS at RT for 10 min. For analysis of eGFP-G3BP expression, cells were directly visualized by confocal fluorescence microscopy. For analysis of cells transfected with pcDNA3/eGFP-G3BP and then infected with ZIKV, fixed cells were permeabilized with nonionic detergent Triton X-100 at the final concentration of 0.1% in PBS. Cells were stained with anti-E mAb 4G2 in PBS containing 1% bovine serum albumin (BSA). Goat anti-mouse IgG Alexa Fluor 594 was used as secondary antibody. The capture of the fluorescent signal was carried out with a confocal fluorescence Nikon Eclipse TI2-S-HU microscope equipped with x63 objectives coupled to the imaging software NIS-Element AR (Nikon, Champigny-sur-Marne, France). Image processing based on FIJI/ImageJ software was performed to estimate the size in μm^2^ of eGFP-positive condensates.

### Flow cytometry

Cells were fixed with 3.7% PFA in PBS and then permeabilized with 0.15% Triton X-100 in PBS. After incubation of cells with a blocking solution for 10 min, cells were stained with mouse anti-E protein mAb 4G2 or rabbit anti-FLAG antibody as primary antibody for 1h. anti-mouse IgG Alexa Fluor 488 or anti-rabbit IgG Alexa Fluor 488 served as secondary antibody. Immunostained cells were subjected to flow cytometric analysis using FACScan flow cytometer (CytoFLEX, Beckman Coulter, Brea, CA, USA). For each assay, at least 10000 cells were analyzed, and the percentage of positive cells was determined using CytExpert software (version 2.1.0.92, Beckman Coulter, Brea, CA, USA).

### Immunoblotting

Cells were lysed with RIPA lysis buffer and clarified cell lysates separated by in-house 12% SDS–PAGE gel. Proteins were transferred onto nitrocellulose NC Protran membrane and after blocking with 5% no-fat dry milk in PBS-Tween for 30 min, the membrane was probed with rabbit anti-FLAG antibody for 1h at room temperature (RT). Anti-rabbit IgG-HRP conjugate was used as secondary antibody. Membranes were then incubated with Pierce ECL Western blotting substrate (Thermo Fisher Scientific) and exposed using Amersham imager 680 (GE Healthcare).

### Cytotoxicity assay

Cell damage was evaluated measuring lactate dehydrogenase (LDH) release. Supernatants of infected cells were recovered and subjected to a cytotoxicity assay, performed using CytoTox 96 non-radioactive cytotoxicity assay (Promega, Madison, WI, USA) according to manufacturer instructions. Absorbance of converted dye was measured at 490 nm (Tecan). Results of LDH activity are presented with subtraction of control values.

### Statistical analysis

All statistical tests were done using the software GraphPad Prism version 10.1.1. Unpaired *t* test and ANOVA were used in this study.

## Results

### Molecular viral clone GUINEA-18 derived from ZIKV-15555

A reverse genetic approach based on the ISA method was successfully used to generate an infectious molecular clone MR766^MC^ of viral strain MR766-NIID [21,22]. The ISA method was used to generate an infectious molecular clone that represents the genomic RNA of viral strain ZIKV-15555 based on viral sequence that has been deposited under GenBank accession number MN025403. Because both 5’ and 3’ termini of viral genome were lacking, the 5’NCR and 3’NCR sequences were completed with nucleotides 1 to 14 and 10721 to 10807 from MR766-NIID genome, respectively. It should be noted that the viral genetic information retrieved on accession number OL41476 was not suitable for the study.

For the ISA method, the overlapping sub-genomic amplicons Z-1^GUINEA-18^, Z-23^GUINEA-18^, and Z-4^GUINEA-18^ were designed to cover full-length genome sequence of ZIKV-15555 (Fig 1). The three GUINEA-18 amplicons were designed so that they can match with Z-1^MR766^, Z-23^MR766^, and Z-4^MR766^ in order to generate chimeric viruses between GUINEA-18 and MR766^MC^ (S1 Fig). The amplicons were amplified by PCR from the respective plasmids and the purified PCR products were transfected into HEK-293T cells. The recovered infectious molecular clones MR766^MR^ and GUINEA-18 were used to infect VeroE6 cells and after two rounds of amplification, P2 virus stocks were used for further studies. Using viral plaque assay on VeroE6 cells as quantitative method of measuring infectious ZIKV (plaque forming units, PFU) (S2 Fig), infectious virus titers of GUINEA-18 and MR766^MC^ reached up to 7 and 8.5 log PFU.mL^-1^ after passage P2, respectively.

### Characterization of the infectious molecular clone GUINEA-18

Polyprotein sequence alignment between GUINEA-18 and MR766^MC^ identified 49 amino acid substitutions (S1 Fig) which are distributed along structural and non-structural proteins (Table 1). Only prM and NS2B proteins were fully conserved between the two viruses.

**Table 1:** Amino acid changes between West African ZIKV strains ZIKV-15555 (Accession n° MN025403) and MR766-NIID (Accession n° LC002520).

protein	position	ZIKV-15555^1^	MR766-NIID^2^	protein	position	ZIKV-15555^1^	MR766-NIID^2^
**C**	5 12	R V	K I	**NS3**	156 174 185 220 251 358 360 483	V A R A I R I D	I T K V T K V G
**E**	152 156 158 283 341 343	I T H R V A	T I Y K I V	**NS4A**	5	D	E
**NS1**	21 92 146 162 191 213 286	V P K I K R H	I S E V R K Y	**NS4B**	11 20 24 26 91 98 180	S K T I F I V	N R A M L M I
**NS2A**	40 42 121 126 150 177 205	A V E V A F M	V I D I P I V	**NS5**	80 301 383 421 444 536 570 640 658 725 839	H F M V K R A H D H K	Y L I E R K K T N Y T

^1^ Accession n° MN025403

^2^ Accession n° LC002520

**Fig 2:**
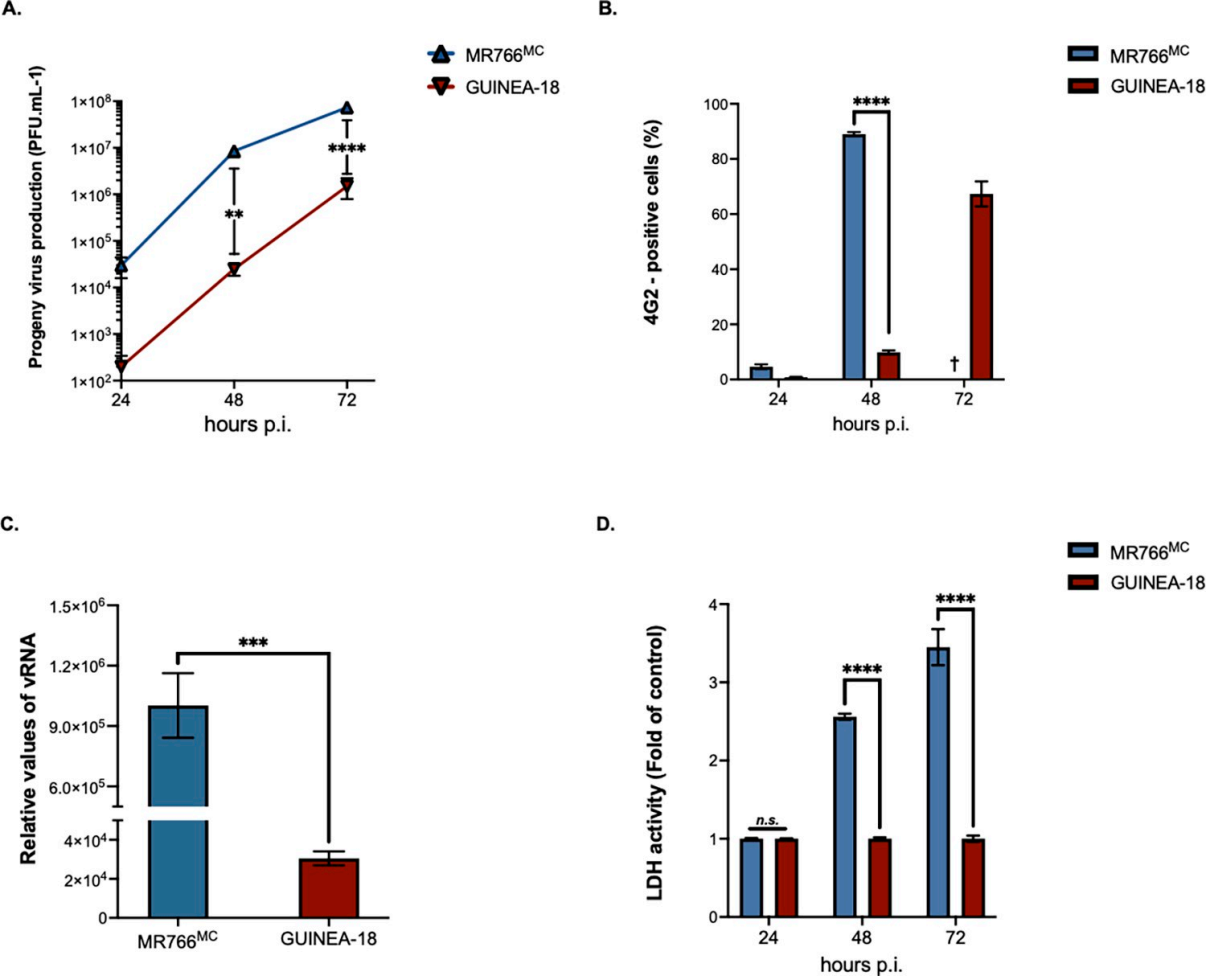
GUINEA-18 replication in non-human fibroblastic VeroE6 cells. Green monkey kidney fibroblastic VeroE6 cells were infected with MR766^MC^ and GUINEA-18 at m.o.i of 0.1 (**A** to **C**) or 1 PFU/cell (**D**). *In* (**A**), virus progeny productions at 24, 48 and 72 h p.i. were quantified by a standard plaque-forming assay. *In* (**B**), FACS analysis was performed on ZIKV-infected cells using anti-*pan* orthoflavivirus E mAb 4G2 and the percentage of 4G2-positive cells was determined. *In* (**C**), intracellular viral RNA production was determined by RT-qPCR at 48 h p.i. RPLPO36B4 mRNA served as a house-keeping RNA control for normalization of samples. *In* (**D**), LDH activity was measured at 24, 48 and 72 h p.i. and expressed as a percentage relative to control. The results are the mean (± SEM) of three independent experiments. Asterisks indicate that the differences between experimental samples at each time point are statistically significant, using the unpaired *t* test and one-way ANOVA (**** *p* < 0.0001, *** *p* < 0.001, ** *p* < 0.01; *n*.*s*.: not significant).

The GUINEA-18 and MR766^MC^ E proteins differ by six amino-acid substitutions with the cluster of mutations I152T/T156I/H158Y that are part of ZIKV glycan loop (GL) region (Table 1). The GL region of GUINEA-18 but not MR766^MC^ may be N-glycosylated to residue E-N154 [27]. Immunoblot assay using anti-E mAb 4G2 showed that GUINEA-18 E protein migrated slower than MR766^MC^ E protein consistent with a glycan potentially linked to GUINEA-18 E-N154 residue (S1 Fig). To analyze the replication of GUINEA-18, VeroE6 cells were infected at a multiplicity of infection (m.o.i) of 0.1 PFU/cell and progeny virus production examined at various times post-infection (p.i.) (Fig 2A). Infection with MR766^MC^ served as control. In comparison with MR766^MC^, virus progeny production of GUINEA-18 was significantly reduced by 3 log at 48 h p.i. Infectious virus titers for GUINEA-18 peaked at 6 log PFU.mL^-1^ at 72 h p.i.. At this time point post infection, extensive cell death was observed in VeroE6 cells infected by MR766^MC^ but not GUINEA-18. The reduced virus production of GUINEA-18 was associated with a significant delay in production level of E protein (Fig 2B). At 48 h p.i., there was a 30-fold reduction in intracellular viral RNA (vRNA) production in VeroE6 cells infected with GUINEA-18 as compared with MR766^MC^ (Fig 2C). At higher m.o.i of infection, GUINEA-18 virus production was still reduced by 2 log at 48 h p.i. and this was associated with lower levels of E protein compared with MR766^MC^ (S4 Fig). A lactate dehydrogenase (LDH) assay was used as indicator of cell damage in ZIKV-infected VeroE6 cells. Viability of VeroE6 cells infected with GUINEA-18 was preserved until 72h p.i. whereas MR766^MC^ caused extensive cell death early on (Fig 2D). Our data indicated that GUINEA-18 was replicating more slowly, and to lower virus production, in VeroE6 cells compared to MR766^MC^.

**Fig 3:**
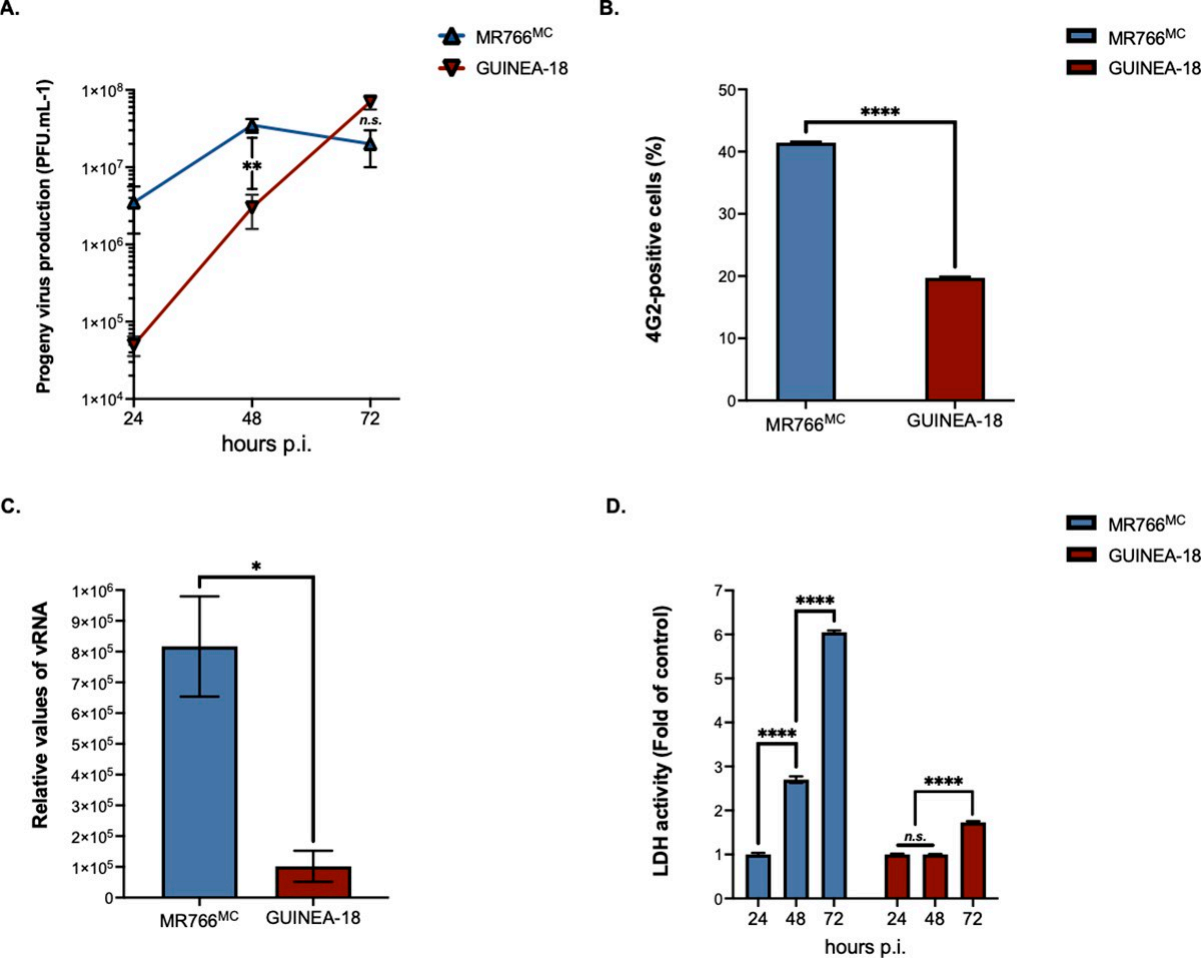
GUINEA-18 replication in human epithelial A549 cells. A549 cells were infected with MR766^MC^ and GUINEA-18 at m.o.i of 1. *In* (**A**), viral progeny production was determined at various times post-infection. *In* (**B**), percentages of ZIKV-infected cells at 48 h p.i. were determined by FACS analysis using anti-E mAb 4G2 as primary antibody. *In* (**C**), intracellular viral RNA production was determined by RT-qPCR at 48 h p.i. RPLPO36B4 mRNA served as a house-keeping RNA control for normalization of samples. *In* (**D**), LDH activity was measured at 24, 48 and 72 h p.i. and expressed as a percentage relative to mock-infected cells (control). The results are the mean (± SEM) of three independent experiments. Asterisks indicate that the differences between experimental samples at each time point are statistically significant, using the unpaired *t* test and one-way ANOVA (**** *p* < 0.0001, ** *p* < 0.01, ** *p* < 0.05; *n*.*s*.: not significant).

The replication properties of GUINEA-18 were first assessed in human cells that have been shown to be permissive to ZIKV infection. Firstly, A549 cells were infected with GUINEA-18 or MR766^MC^ at an m.o.i. of 1. Analysis of viral growth identified a peak of virus production reaching about of 7.5 log mL^-1^ in A549 cells infected for 72 h with GUINEA-18 (Fig 3A). MR766^MC^ infection for 48 h was required to give a similar infectious virus titer. At this timepoint, GUINEA-18 virus production was one 1 log lower compared to MR766^MC^, alongside a 2-fold reduction in E protein expression levels (Fig 3B) and 8-fold reduction in vRNA production (Fig 3C). Cytopathic effects were observed in MR766^MC^-infected A549 cells from 48 h p.i. whereas only a weak loss of cell viability was observed with GUINEA-18 at 72 h p.i. (Fig 3D). These results show that A549 cells are less permissiveness to GUINEA-18 than MR76^MC^. Reduced replication of GUINEA-18 was also observed in HCM3 cells (S5 Fig).

**Fig 4:**
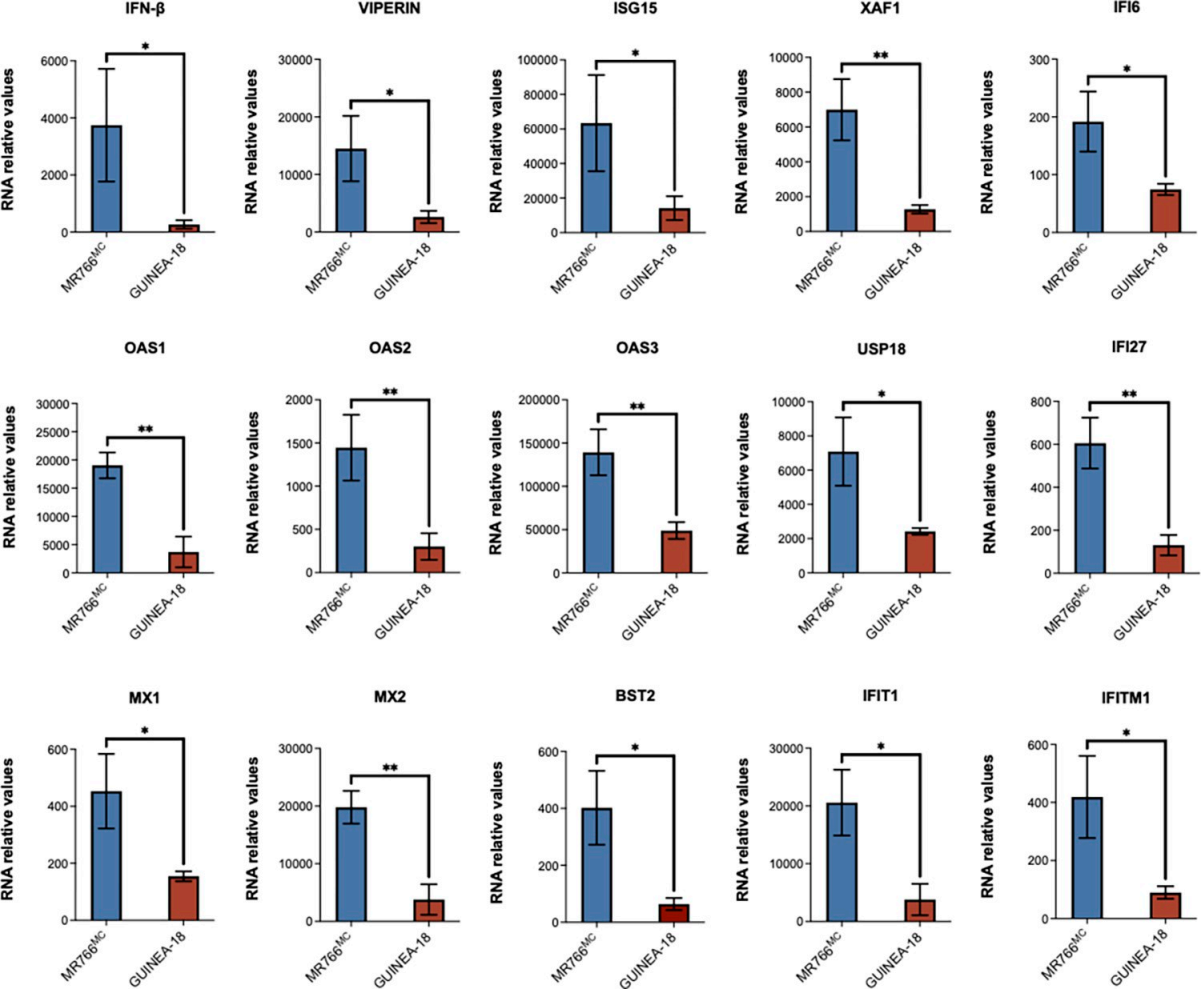
Expression of ISG and IFN-β mRNA in A549 cells infected by ZIKV. A549 cells were infected with GUINEA-18 or MR766^MC^ at an m.o.i. of 1. The relative abundance of IFN-β and ISG mRNA was determined at 48 h p.i. by RT-qPCR. RPLPO36B4 mRNA served as a house-keeping RNA control for normalization of samples. Results are expressed as the fold-induction of IFN-β and ISG mRNA in ZIKV-infected cells relative to those in mock-infected cells. The results are the mean (± SEM) of three independent experiments. Asterisks indicate that the differences between experimental samples at each time point are statistically significant, using the unpaired *t* test (** *p* < 0.01, * *p* < 0.05).

Next, we evaluated whether the lower replication capacity of GUINEA-18 in A549 cells was associated to a change in activation of antiviral innate immune responses when compared to MR766^MC^. ZIKV is inhibited by type I IFNs such as IFN-β and antiviral effector proteins, so called interferon-stimulated genes (ISGs) following recognition of viral nucleic acids and molecular features associated with viral infection of host cells [26,28–31]. The relative expression of ISGs and IFN-β mRNA was assessed by RT-qPCR on total RNA isolated from ZIKV-infected A549 cells at 48 h p.i. (Fig 4). As evidenced by analysis of fourteen representative ISGs, ISG mRNA levels were significantly lower in A549 cells infected by GUINEA-18 compared to MR766^MC^. As a control of ISG protein production, ISG15 was weakly detected in A549 cells infected by GUINEA-18 compared with MR766^MC^ (S6 Fig). Moreover, mRNA levels of IFN-β was also much lower in A549 cells infected by GUINEA-18 in comparison with MR766^MC^. Thus, infection of A549 cells with GUINEA-18 induced lower ISG and IFN-β responses than MR766^MC^.

### Role of the 5’ and 3’ regions in GUINEA-18 replication

We next examined whether the reduced replicative abilities of GUINEA-18 depended on virus-specific determinants. A chimeric GUINEA-18 virus in which the Z-1^GUINEA-18^ amplicon was swapped for Z-1^MR766^ was used to assess the importance of 5’ region in replication properties of GUINEA-18 (Fig 1B). The resulting GUINEA-18/MR766^[5’region]^ virus includes the 5’NCR mutations G19A and C41T and eight amino-acid substitutions that are distributed between C [2] and E [6] proteins (Fig 1). Of note, mutation E-T156I removes the N-glycosylation site of the E protein, resulting in a non-glycosylated chimeric virus [22,32]. A549 cells were infected with chimeric GUINEA-18/MR766^[5’region]^ virus or GUINEA-18 at an m.o.i. of 1 (S7 Fig); with infection by MR766^MC^ as control. At 48 h p.i., there was no significant change in virus production between GUINEA-18/MR766^[5’region]^ and GUINEA-18 (S7A Fig). There were also similar expression levels of E protein (S7B Fig). These results do not argue in favor of major role of the 5’NCR and structural proteins in the replication properties of GUINEA-18.

We next addressed the role of 3’ region of viral genome in the replicative abilities of GUINEA-18. A chimeric MR766^MC^ virus was obtained in which Z-4^MR766^ amplicon was replaced by Z-4^GUINEA-18^ leading to a swapping of both NS5 and 3’NCR sequences between MR766^MC^ and GUINEA-18 (Fig 1B). There are eleven amino-acid substitutions that are distributed between MTase and RdRp domains of the NS5 protein (Table 1). Eight mutations differentiate the 3’NCRs from GUINEA-18 and MR766^MC^ (S8A Fig). The most notable mutations in SLII and DB structures might have an effect on viral RNA replication (S8B Fig) [33]. Transfection with Z-1^MR766^, Z-23^MR766^, and Z-4^GUINEA-18^ amplicons resulted in virus production with an infectious titer of 3 log PFU.mL^-1^ at passage 2 on VeroE6 cells. Thus, the swapping of Z-4^GUINEA-18^ amplicon into MR766^MC^ restrained viral replication by at least 5 log compared with MR766^MC^. To determine whether the inability of chimeric MR766/GUINEA-18^[3’region]^ virus to replicate depended on the 3’NCR, site-directed mutagenesis was conducted on Z-4^MR766^ amplicon to generate a mutant MR766/GUINEA-18^[3’NCR]^ virus bearing all GUINEA-18 3’NCR mutations (Fig 1 and S8A). The mutant virus was assessed in VeroE6 cells (S9 Fig). At 48 h p.i., there was comparable virus production between MR766/GUINEA-18^[3’NCR]^ and MR766^MC^ (S8A Fig). This was associated with a similar percentage of ZIKV-infected cells (S9B Fig). The mutant virus was more cytopathic in VeroE6 cells than the parent (S9C Fig). These results rule out a role for the 3’NCR in the replicative abilities of a chimeric MR766^MC^ virus with the 3’ region of GUINEA-18. Our data indicated that a chimeric MR766^MC^ virus with GUINEA-18 NS5 protein is unable to sustain infection of mammalian cells, precluding the study of the largest NS protein in attenuation of GUINEA-18.

### The replicative properties of GUINEA-18 depend on NS1 to NS4B proteins

**Fig 5:**
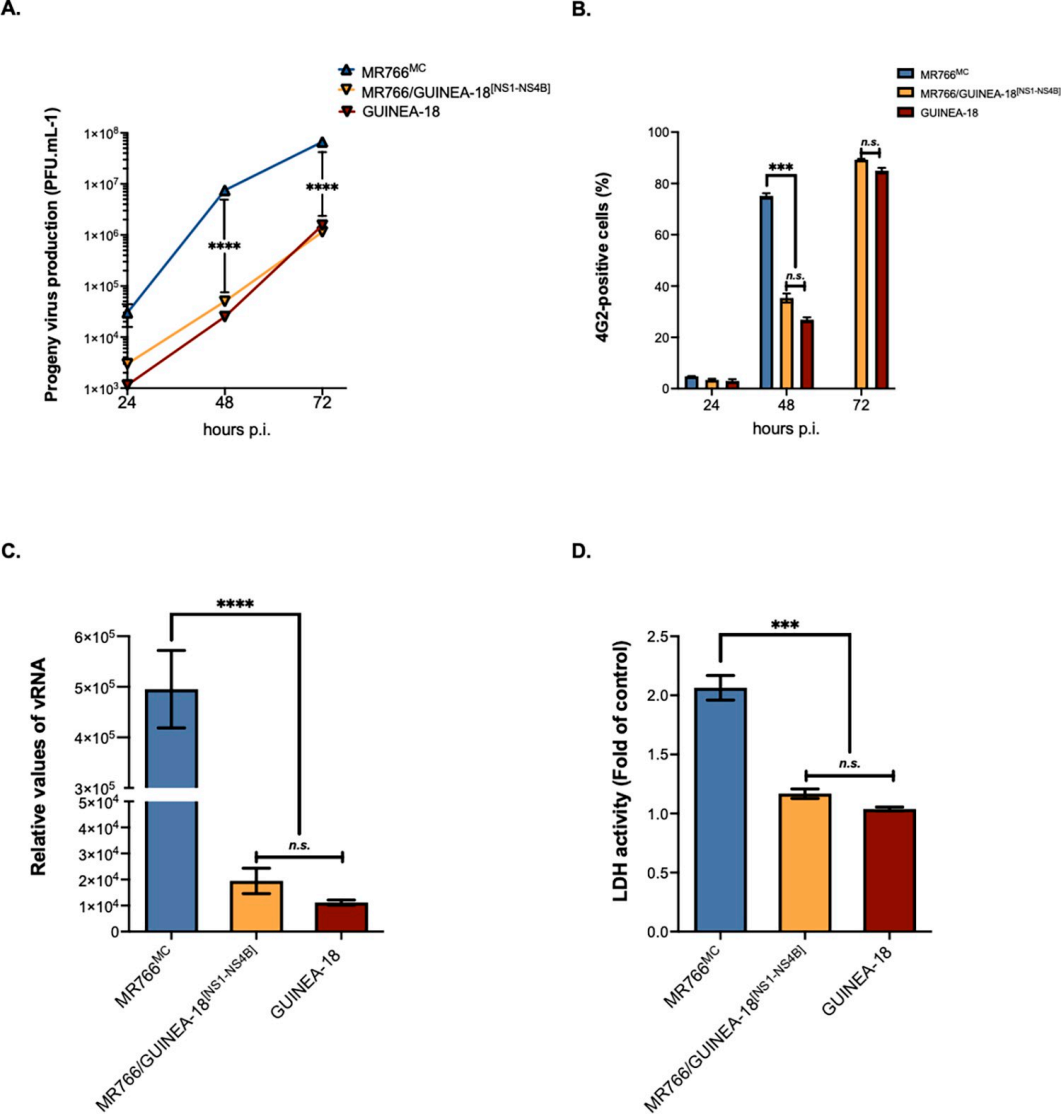
MR766/GUINEA-18^[NS1-NS4B]^ replication in VeroE6 cells. VeroE6 cells were infected with MR766/GUINEA-18^[NS1-NS4B]^ chimeric virus or parental viruses at m.o.i of 0.1. *In* (**A**), virus progeny production at 24h, 48h, and 72h. *In* (**B**), percentage of ZIKV-infected cells at 24h, 48h, and 72h measured by FACS analysis using mAb 4G2. *In* (**C**), RT-qPCR was performed on viral RNA (vRNA) extracted from VeroE6 cells infected for 48h with ZIKV. RPLPO36B4 mRNA served as a house-keeping RNA control for normalization of samples. *In* (**D**), LDH activity was measured at 72 h p.i. The results are the mean (± SEM) of three independent experiments. Asterisks indicate that the differences between experimental samples at each time point are statistically significant, using the unpaired *t* test and one-way ANOVA (**** *p* < 0.0001, *** *p* < 0.001; *n*.*s*.: not significant).

To evaluate the importance of NS1 to NS4B proteins in the replication of GUINEA-18, a chimeric MR766/GUINEA^[NS1-NS4B]^ virus was generated, in which the MR766^MC^ region coding for NS1 to NS4B proteins was swapped with their counterpart in GUINEA-18 (Fig 1C). The chimeric MR766/GUINEA^[NS1-NS4B]^ virus was compared with MR766^MC^ in VeroE6 cells infected for 48 h at an m.o.i. of 0.1 (Fig 5). Infection with GUINEA-18 served as control. Analysis of viral growth showed that insertion of GUINEA-18 NS1 to NS4B into MR766^MC^ strongly reduced virus production (Fig 5A). Virus production was similar between MR766/GUINEA-18^[NS1-NS4B]^ and GUINEA-18 (Fig 5A), and the two viruses were comparable for the E protein expression level (Fig 5B) and intracellular vRNA production in VeroE6 cells (Fig 5C). Late in infection, chimeric MR766/GUINEA-18^[NS1-NS4B]^ virus preserved cell viability, as observed for GUINEA-18 (Fig 5D). Our data thus indicated a key role for NS1 to NS4B proteins in the replication properties of GUINEA-18.

**Fig 6:**
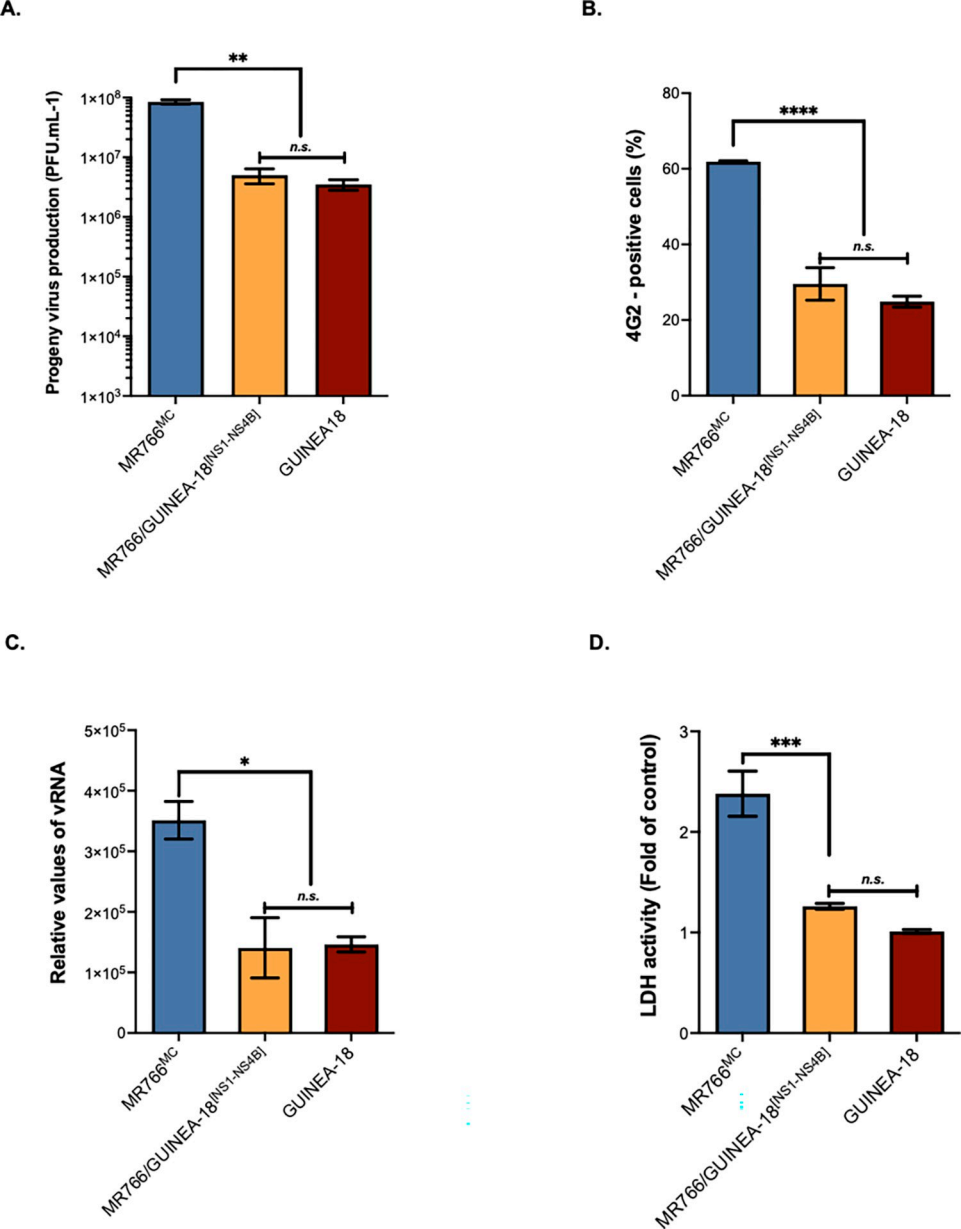
MR766/GUINEA-18^[NS1-NS4B]^ replication in A549 cells. A549 cells were infected for 48h with MR766/GUINEA-18^[NS1-NS4B]^ chimera or parental viruses at m.o.i of 1. *In* (**A**), virus progeny production. *In* (***B***), RT-qPCR was performed on viral RNA (vRNA) extracted from ZIKV-infected VeroE6 cell. RPLPO36B4 mRNA served as a house-keeping RNA control for normalization of samples. *In* (**C**), percentage of ZIKV-infected cells measured by FACS analysis using mAb 4G2. *In* (**D**), LDH activity was measured at 48 h p.i. The results are the mean (± SEM) of three independent. Asterisks indicate that the differences between experimental samples at each time point are statistically significant, using the unpaired *t* test and one-way ANOVA (**** *p* < 0.0001, ** *p* < 0.01; * *P* < 0.05; *n*.*s*.: not significant).

The replication efficiency of the MR766^MC^ chimera with GUINEA-18 NS1 to NS4B proteins was next assessed in A549 cells (Fig 6). The behavior of chimeric MR766/GUINEA-18^[NS1-NS4B]^ virus was indistinguishable from GUINEA-18 in viral replication (Fig 6A–6C) and cytotoxicity (Fig 6D) reinforcing the notion that NS1 to NS4B proteins influence GUINEA-18 replication in mammalian host cells.

**Fig 7:**
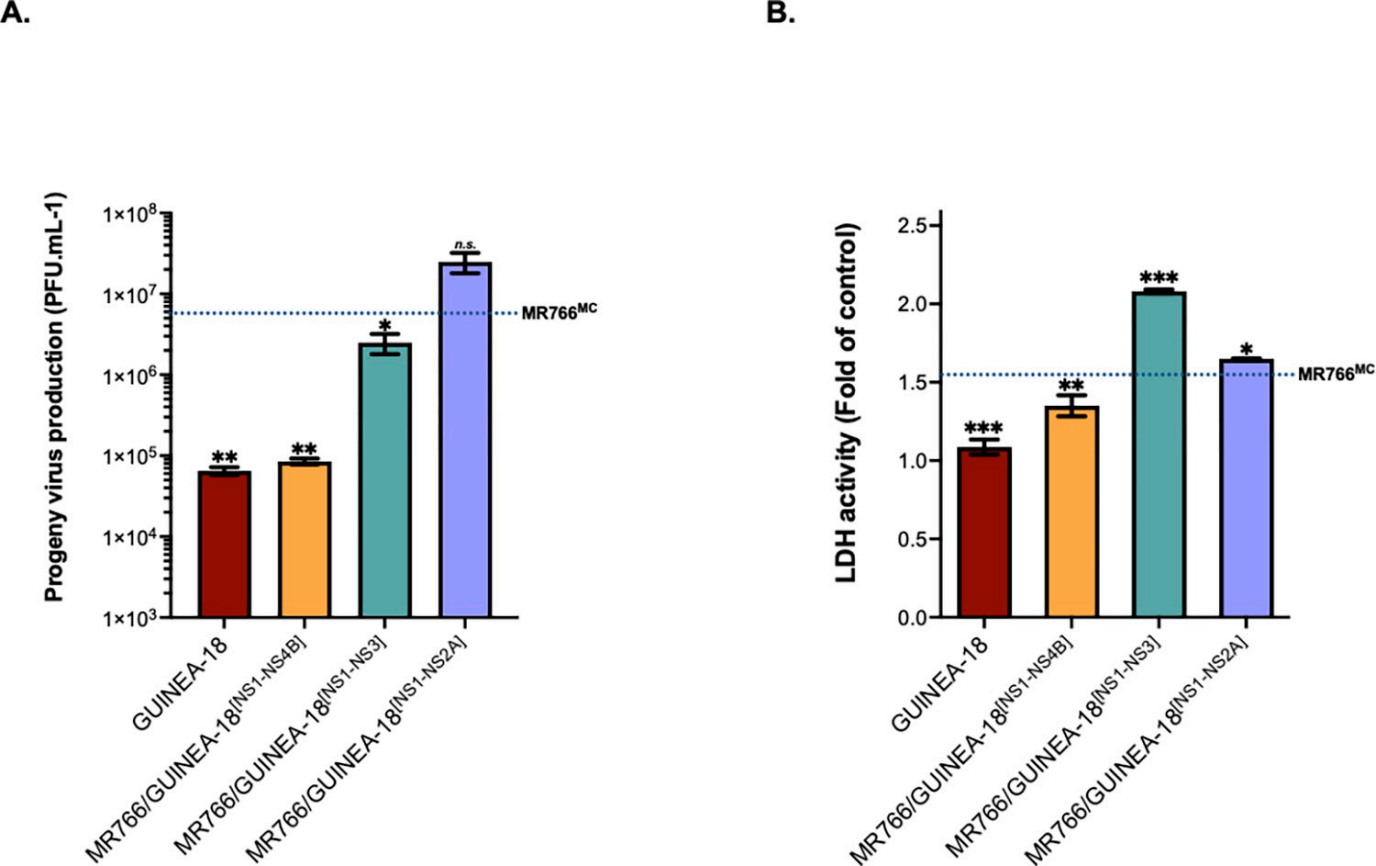
Effects of GUINEA-18 NS proteins on MR766^MC^ replication. VeroE6 cells were infected with MR766/GUINEA-18 chimeric viruses containing GUINEA-18 NS proteins or parental ZIKV at an m.o.i. of 0.1. *In* (**A**), virus progeny production at 48h p.i. The dotted line indicates MR766^MC^ progeny production. *In* (**B**), LDH activity was measured at 72 h p.i. The dotted line indicates the rate of LDH release with MR766^MC^. The results are the mean (± SEM) of three independent experiments. Statistical analysis relative to MR766^MC^ values was noted. Asterisks indicate that the differences between experimental samples at each time point are statistically significant, using the unpaired *t* test (*** *p* < 0.001, ** *p* < 0.01, * *p* < 0.05; *n*.*s*.: not significant).

To better understand the impact of NS1 to NS4B proteins on GUINEA-18 replication properties, we produced two additional chimeric viruses, MR766/GUINEA-18^[NS1-NS3]^ and MR766/GUINEA-18^[NS1-N2A]^, containing GUINEA-18 NS1/NS2AB/NS3 or NS1/NS2A in an MR766^MC^ backbone, respectively (Fig 1C). The amino-acid sequence of NS2B protein is conserved between GUINEA-18 and MR766^MC^ (S1 Fig). The replication of chimeric viruses was analyzed in VeroE6 cells infected at an m.o.i. of 0.1 and compared with parental viral clones (Fig 7A). Infection with MR766/GUINEA^[NS1-NS4B]^ chimera served as control. Analysis of virus production revealed that MR766^MC^ chimeric viruses with GUINEA-18 NS1-NS3 or NS1-NS2A proteins have little or no effect on virus replication (Fig 7A). Infection with chimeric MR766^MC^ viruses resulted in extensive cell death (Fig 7B). Thus, the swapping of NS1 to NS3 proteins was not sufficient to affect MR766^MC^ replication and cytotoxicity emphasizing the major role of NS4B in the attenuated phenotype of GUINEA-18 *in vitro*.

The above results suggest that the replicative properties of GUINEA-18 depend on NS4B protein. Among seven amino-acid substitutions that differentiate NS4B of ZIKV-1555 and MR766, four have been identified at positions 11, 20, 24 and 26 (Table 1). The ZIKV-15555 residues R20 and T24 have been also identified in West African ZIKV strains Senegal-Kedougou 2011 and Senegal-Kedougou 2015 (European Nucleotide Archive accession number n°PRJEB39677) that have been isolated from mosquito pools in Casamance region of Senegal in 2011 and 2015, respectively [19]. Residues R20 and T24 were not observed in Asian and Asian-related American viruses. A change from non-polar amino-acid alanine to polar residue threonine at position 24 might influence NS4B conformation. A three-dimensional structure of the N-terminal region of NS4B was performed by modelling on Phyre^2^ [34] allowing *de novo* peptide structure prediction (S10A Fig). Structural analysis showed that ZIKV-1555 NS4B residues 1 to 16 and 35 to 50 have propensity for forming a helical structure and transmembrane helix, respectively. The NS4B amino-acid substitutions at positions 20, 24, and 26 that differentiate ZIKV-15555 from MR766 have been identified in a non-ordered structure between helix α1 and TM1 [35].

**Fig 8:**
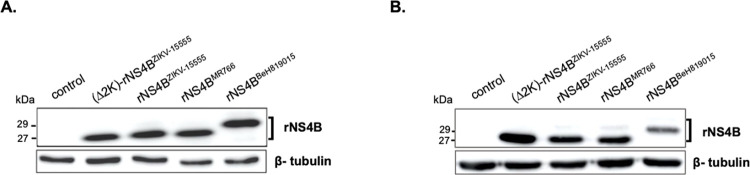
Intracellular expression of recombinant ZIKV NS4B protein. A549 (**A**) and HCM3 (**B**) cells were transfected for 24 h with pcDNA3 expressing rNS4B protein from ZIKV-15555 (rNS4B^ZIKV-15555^), MR766 (rNS4B^MR766^), or BeH819015 (rNS4B^BeH819015^). The rNS4B^ZIK-15555^ protein mutant lacking the 2K peptide is indicated as (Δ2K)-rNS4B^ZIK-15555^. Cell lysates in RIPA buffer were assessed by immunoblot assays with anti-epitope antibody FLAG as the primary antibody. The β-tubulin protein served as a protein-loading control. The positions of rNS4B are indicated.

We assessed whether the NS4B mutations that differentiate ZIKV-15555 from MR766 impact protein expression. Recombinant 2K-NS4B (hereafter intitled rNS4B) proteins derived from ZIKV-15555, MR766, and BeH819015 were expressed in A549 cells using from pcDNA3. The rNS4B proteins were C-terminally tagged with FLAG epitope. The ZIKV-15555 rNS4B protein expressed without the 2K peptide served as control. Next, rNS4B protein expression in transfected A549 cells was verified by FACS analysis using anti-FLAG antibody (S11 Fig). Levels of rNS4B protein expression were comparable across proteins expressed in A549 cells (Fig 8). Immunoblot assays using anti-FLAG antibody allowed the detection of MR766 and ZIKV-15555 rNS4B proteins in A549 (Fig 8A) and HCM3 cells (Fig 8B). Migration was comparable with an apparent molecular weight similar to rNS4B mutant without 2K indicating that the N-terminal peptide was correctly processed from the N-terminus of protein. The change in migration profile of BeH819015 NS4B protein compared to ZIKV-15555 and MR766 could be due to specific residues that have been identified in epidemic Asian/American ZIKV strains (S10B Fig). We can conclude that the seven NS4B amino-acid substitutions that differentiate ZIKV-15555 from MR766 have no obvious effect on protein expression.

**Fig 9:**
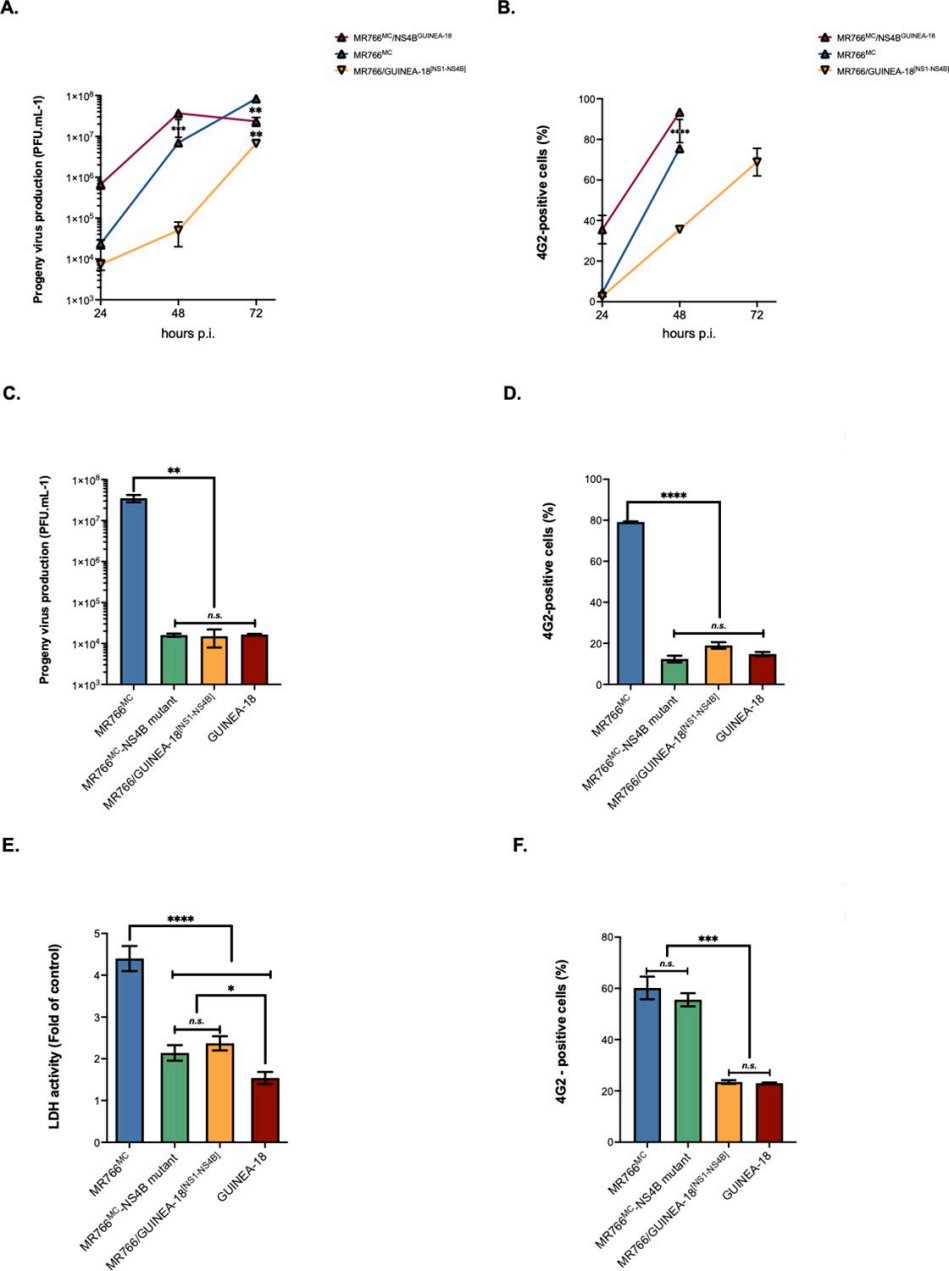
Effect of GUINEA-18 NS4B on MR766^MC^ replication. VeroE6 cells (**A-E**) and A549 cells (**F**) were infected with GUINEA-18, MR766^M^, MR766^MC^ mutant containing the GUINEA-18 NS4B residues S11/R20/T24/I26/V180 (MR766^MC^-NS4B mutant) or MR766^MC^ chimeric virus containing the GUINEA-18 NS4B protein (MR766^MC^/NS4B^GUINEA-18^). MR766/GUINEA-18^[NS1-NS4B]^ served as a control. *In* (**A-B**), VeroE6 cells were infected with MR766^MC^/NS4B^GUINEA-18^ at m.o.i 0.1. Virus progeny production (**A**), and percentage of ZIKV-infected cells measured by FACS analysis using mAb 4G2 (**B**). *In* (**C-E**), VeroE6 cells were infected with MR766^MC^-NS4B mutant at m.o.i 0.1. Virus progeny production (**C**), and percentage of ZIKV-infected cells measured by FACS analysis using mAb 4G2 (**D**) were determined at 48 h p.i. LDH activity was measured at 72 h p.i. **(E).** *In* (**F**), A549 cells were infected with MR766^MC^-NS4B mutant at m.o.i 1. The percentages of ZIKV-infected cells measured by FACS analysis using mAb 4G2 were determined at 48 h p.i. The results are the mean (± SEM) of two or three independent experiments. Asterisks indicate that the differences between experimental samples at each time point are statistically significant, using the unpaired *t* test and one-way ANOVA (**** *p* < 0.0001, *** *p* < 0.001, ** *p* < 0.01; * *p* < 0.05; *n*.*s*.: not significant).

To better understand the role of NS4B in GUINEA-18 replication, the NS4B gene of MR766^MC^ was mutated to introduce the seven amino-acid substitutions of GUINEA-18 NS4B protein (Fig 1C). Because most of the mutations have been identified in the N-terminal region of NS4B protein (Table 1), we produced a mutant MR766^MC^ virus with the S11/R20/T24/I26/V180 residues. The chimeric MR766^MC^/NS4B^GUINEA-18^ and mutant MR766^MC^-NS4B viruses were assessed for replication in VeroE6 cells at an m.o.i. of 0.1 (Fig 9). Parental viruses and chimeric MR766/GUINEA-18^[NS1-NS4B]^ virus served as controls. Analysis of MR766^MC^/NS4B^GUINEA-18^ chimera revealed that insertion of GUINEA-18 NS4B in MR766^MC^ caused no reduction in virus production (Fig 9A) and no effect on expression level of E protein (Fig 9B). By contrast, infection with mutant MR766^MC^-NS4B virus resulted in low virus production, comparable to that observed with GUINEA-18 or MR766/GUINEA-18^[NS1-NS4B]^ (Fig 9C). This correlated with weak expression levels of E protein (Fig 9D). Viability of VeroE6 cells infected with MR766^MC^-NS4B mutant was preserved at 72 h p.i. (Fig 9E). The effects of GUINEA-18 NS4B mutations on MR766^MC^ were not observed in A549 cells raising the possibility of a cellular context dependent effect of the N-terminal NS4B residues on virus replication (Fig 9F).

### GUINEA-18 NS1 to NS4B proteins influence stress granule formation

In response to RNA virus infection, cellular stress granules (SGs) are assembled as cytoplasmic condensates which can sequester viral RNA and proteins thus restricting viral growth [36–38]. A common feature of infection with orthoflaviviruses including ZIKV is inhibition of SG assembly facilitating viral replication and limiting antiviral signaling activation in infected host-cells [39–41]. In particular, ZIKV hijacks the core SG proteins such as RasGAP SH3 domain-binding protein (G3BP) [40]. Other studies supported the idea that ZIKV uses NS proteins to interfere on SG formation [28]. In this regard, we were wondering whether GUINEA-18 NS1 to NS4B proteins might differ from MR766^MC^ NS proteins in their ability to inhibit SG assembly in infected cells. To investigate this further, A549 cells were infected with chimeric MR766^MC^/GUINEA-18^[NS1-NS4B]^ virus at an m.o.i. of 2. Both MR766^MC^ and GUINEA-18 were used as controls. Prior to virus infection, A549 cells were transfected with a plasmid expressing eGFP reporter fused in-frame to the N-terminus of G3BP which is considered a SG marker [42,43]. SG formation was examined in ZIKV-infected A549 cells under environmental stress. Sorbitol as a physiological osmotic and oxidative stressor was used to drive GFP-G3BP fusion protein to SGs [44,45]. By confocal fluorescence microscopy, cytoplasmic eGFP-positive condensates with an average surface area of 2.5 μm^2^ were observed in A549 cells incubated with 0.4M sorbitol for 1.5 h (S12 Fig).

**Fig 10:**
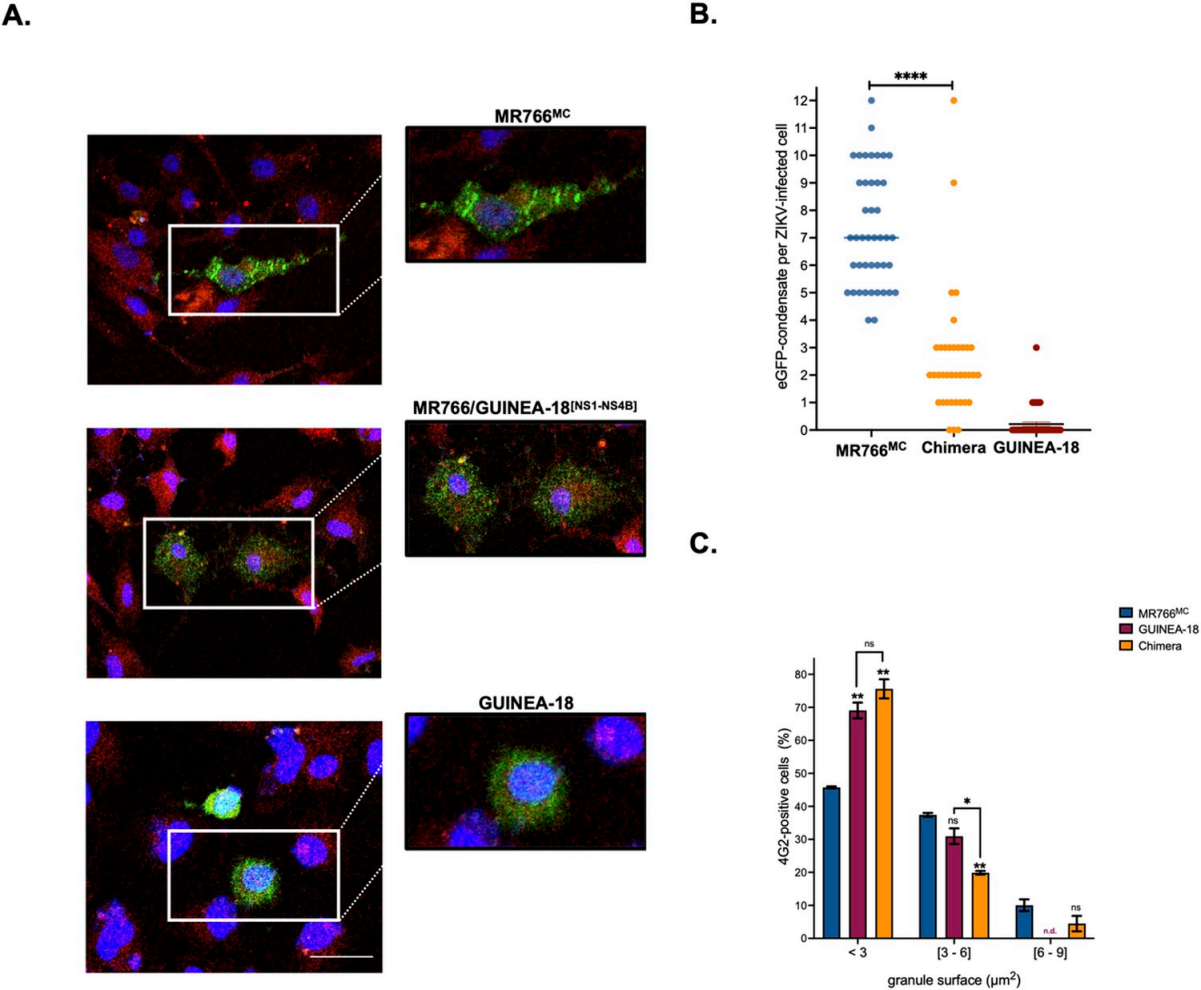
Stress granule formation in ZIKV-infected cells stressed with sorbitol. A549 cells were transfected with pcDNA3/eGFP-G3BP for 6 h and then infected with ZIKV at an m.o.i. of 2. At 40 h p.i., cells were stressed with 0.4 M sorbitol for 1.5 h. *In* (**A**), cells infected with MR766^MC^, GUINEA-18, or MR766/GUINEA-18^[NS1-NS4B]^ chimera were identified using anti-E mAb 4G2. Cells positive for ZIKV E (red) and eGFP-G3BP (green) proteins were examined by confocal microscopy analysis. Nuclei were stained with DAPI (blue). Scale bar, 50 μm. *In* (**B**), cells positive for E protein expression (n ~ 50) were scored for the number of eGFP-positive condensates per ZIKV-infected cell. Values from 4 independent experiments are presented. Asterisks indicate that the differences between experimental samples at each time point are statistically significant, using 2-way ANOVA test (**** *p* < 0.0001). *In* (**C**), cells positive for E protein and eGFP-G3BP expression (n ~ 100) were scored for the surface of EGFP-positive condensates as small (< 3 μm^2^), medium (3–6 μm^2^) and large (6–9 μm^2^) defined granules. Large SGs were not detected (n.d.) in A549 cells infected by GUINEA-18. Values from two independent experiments are presented. Asterisks indicate that the differences between experimental samples at each time point are statistically significant, using the unpaired *t* test (** *p* < 0.01; * *p* < 0.05; ns: not significant).

The abundance of eGFP-positive condensates was determined in A549 cells infected with ZIKV for 40 h and then stressed with 0.4M sorbitol for 1.5 h (Fig 10). Infection by ZIKV was confirmed by IF assay using anti-E mAb 4G2. Confocal fluorescence microscopy detected eGFP-positive condensates in A549 cells infected by MR766^MC^ (Fig 10A). Almost fifty 4G2-positive cells were scored for the number of eGFP-positive condensates per cell profile. An average of seven defined granules per cell was observed in A549 cells infected by MR766^MC^ (Fig 10B). Almost 50% of the eGFP-positive condensates had a surface area ranging from 3 to 9 μm^2^ with an average of approximately 4.1 μm^2^ (Fig 10C). As assessed by quantifying eGFP-positive condensates, we observed that GUINEA-18 actively blocked SG formation under environmental stress (Fig 10B). Almost 80% of granules had surface area lower than 3 μm^2^ (Fig 10C). Thus, GUINEA-18 showed greater efficiency to prevent SG formation in A59 cells stressed with sorbitol, compared to MR766^MC.^

Infection with chimeric MR766/GUINEA^[NS1-NS4B]^ virus resulted in a weak number of eGFP-positive condensates in A549 cells (Fig 10A). The abundance of defined granules was reduced by 50% compared to parental virus (Fig 10B). The surface area of eGFP-positive condensates was approximately 2.5 μm^2^ (Fig 10C). Our data using chimeric MR766/GUINEA^[NS1-NS4B]^ virus suggest that NS1 to NS4B proteins may play an essential role in inhibition of ZIKV-mediated SG formation. We conclude that GUINEA-18 NS proteins have evolved mechanisms to restrain SG formation, with possible implications for viral propagation.

## Discussion

Most studies aiming to understand the transmission, biology, and pathogenicity of emerging ZIKV are still carried out using epidemic strains of Asian genotype. Little information is available on the replication properties of contemporary African ZIKV strains, despite their ability to be transmitted by *Aedes* mosquitoes and their teratogenic potential for humans. It has been proposed that the greater level of pathogenicity of African ZIKV strains might be the cause of fetal loss rather than neurodevelopmental malformations [19]. This could explain the difficulties to estimate the adverse pregnancy outcomes associated with ZIKV infection in sub-Saharan Africa [46–48].

It is critical to improve our knowledge on features of recently isolated ZIKV strains from West Africa. Genomic RNA of viral strain ZIKV-15555 sequenced from an individual who had been exposed to ZIKV in the Republic of Guinee (Faranha region) in 2018 has been considered as suitable for studying contemporary West Africa viral strains. Here, we developed an infectious molecular clone GUINEA-2018 that has been obtained from ZIKV-15555 RNA sequence by reverse genetics. MR766^MC^ from historical African ZIKV strain MR766 was chosen as prototype model of ZIKV of African genotype in our study [22]. Polyproteins of viral clones GUINEA-18 and MR766^MC^ share a high degree of amino-acid homology (98.6% of identity) with only six amino-acid substitutions in the E protein. Also, there were a limited number of nucleotide mutations between the 5’NCR and 3’NCR of the two viral sequences. Cell cultures studies of GUINEA-18 replication were conducted in comparison with MR766^MC^. Viral growth analysis showed that GUINEA-18 replication is markedly attenuated in VeroE6 cells compared to MR766^MC^. Attenuation of GUINEA-18 coincided with low vRNA production rate and reduced production of intracellular E protein. Cell viability was mostly preserved during GUINEA-18 infection whereas extensive cell death occurred with MR766^MC^ within the three days. The replicative ability of GUINEA-18 was also attenuated in A549 cells, leading to a slight loss of cell viability. Infection with GUINEA-18 resulted to a moderate up-regulation of IFN-β and ISGs expression in A549 cells when compared with MR766^MC^. The weak induction of innate immune responses might be a direct consequence of GUINEA-18 replication. Alternatively, GUINEA-18 may have evolved mechanisms to blunt antiviral defenses to benefit viral propagation.

In an effort to identify genetic determinants involved in the properties of GUINEA-18, chimeric viruses were generated by interchanging viral sequences from these two African ZIKV strains. Two mutations have been identified between the 5’ NCR of GUINEA-18 and MRC766^MC^. Their structural protein regions differ by only 2 and 6 amino acid substitutions in the C and E proteins, respectively. The prM protein which has been proposed as viral factor playing a key role in the neuropathogenicity of epidemic Asian ZIKV strains was unchanged. GUINEA-18 E protein includes a flexible GL (residues E-145 to E-164) region where a glycan is linked to residue N154 [27]. The N-glycosylation of the GL region has been also observed with epidemic ZIKV strains of Asian genotype [27]. In contrast, MR766^MC^ carries the T156I substitution which abrogates the N-glycosylation site resulting in a non-glycosylated E protein [27]. Replacing of GUINEA-18 structural protein region by its counterpart from MR766^MC^ generated a chimeric virus showing similarity in replication and cytotoxicity to GUINEA-18. This excludes a role for the C and E proteins in attenuation of GUINEA-18 *in vitro*. The permutation of MR766^MC^ 3’NCR by the counterpart from GUINEA-18 that differs by eight mutations had no effect on MR766^MC^ replication, also precluding a possible role for the 3’end of genomic RNA in the attenuation of GUINEA-18 *in vitro*.

Forty-one amino-acid substitutions have been identified between the NS proteins of ZIKV-15555 and MR766-NIID (Table 1). Only the NS2B protein was unchanged between the two viral strains. The inability of a chimeric MR766^MC^ virus with GUINEA-18 NS5 to sustain productive infection makes it unachievable to evaluate the role for the largest NS protein in GUINEA-18 replication properties. The data using chimeric viruses between MR766^MC^ and GUINEA-18 however assigned an essential role for NS1 to NS4B in GUINEA-18. Among them, NS4B protein was identified as a critical molecular determinant. The N-terminal region of GUINEA-18 NS4B contains the K20 and T24 residues, unique for contemporary West African ZIKV strains. Analysis of a mutant MR766 virus with the N-terminal residues of GUINEA-18 NS4B protein demonstrated a potential effect of K20 and T24 residues on virus replication, depending on cellular environment.

The precise mechanisms that contribute to the attenuated phenotype of GUINEA-18 *in vitro* remain to be addressed. The composition of GUINEA-18 NS proteins might have a direct effect on replicative processes thereby interfering with the ability of virus to hijack innate immune responses [49]. The fact that a chimeric MR766 virus with GUINEA-18 NS5 protein was defective in viral propagation suggests a decrease in affinity of NS5 for the MR766 NS1 to NS4B proteins. Another possibility is that the ZIKV-mediated SG formation blockade may account for the reduced replication efficiency of GUINEA-18. The formation of SGs as biomolecular condensates including G3BP protein is induced by various stresses including viral infections [37,42]. It is still unclear how SGs interfere with RNA virus replication and antiviral innate immunity [50]. Inhibition of SG formation or SG disassembly contributes to strategies by which orthoflaviviruses can hijack innate immunity to their own benefit [39]. In A549 cells infected with ZIKV and then stressed with sorbitol as environmental SG inducer, GUINEA-18 showed a greater efficacy to prevent SG formation than MR766^MC^. Sorbitol-induced SG formation of SGs was greatly affected in A549 cells infected by a chimeric MR766^MC^ carrying GUINEA-18 NS1 to NS4B proteins, emphasizing a potential role for NS proteins in ZIKV- strain specific SG formation blockade. It is still unknown which specific NS protein(s) may be responsible for suppressing SG formation. Further studies focusing on the mechanisms of GUINEA-18-mediated SG formation blockade are needed to better understand the role of NS protein, with a particular emphasis on the NS4B protein. This raises the question of whether the ZIKV-mediated SG formation blockade impacts expression of ISGs and IFN-β in A549 cells infected by GUINEA-18.

In our study, a comparative analysis with historical African ZIKV strain MR766 showed the contemporary African viral strain ZIKV-15555 as attenuated *in vitro*. Our data indicate that this attenuated phenotype of viral strain depends on NS1 to NS4B proteins with a particular emphasis on N-terminal region of NS4B. The specific role of each NS protein on the attenuated phenotype and restricted cytotoxicity of ZIKV-15555 *in vitro* remains to be investigated. It is possible that ZIKV-15555 is more efficient than MR766 in suppressing SG formation, or in facilitating SG disassembly. The weak induction of ISGs in the host-cells infected by ZIKV-15555 may rely to the capacity of virus to interfere with SG formation. It is therefore of priority to determine whether the NS proteins of contemporary African viral strains contribute to ZIKV greater ability to hijack antiviral innate immunity by limiting SG assembly in the host cells that they infect. This might play an important role in the virulence of contemporary Africa viral strain ZIKV-15555 infection, and remains to be elucidated *in vivo* [51].

## Supporting information

S1 Table Sequences of primers for ISA and RT-qPCR used in this study. Forward (F) and Reverse (R) primers.
(DOCX)

S1 Fig ZIKV polyprotein. Alignment of polyproteins (amino-acids 1 to 3423) from viral strains ZIKV-15555 (Accession n° MN025403) and MR766-NIID (Accession n° LC002520). Arrows indicate the starting amino acid of each mature viral protein. The amino-acid substitutions between ZIKV-15555 and MR766-NIID - are indicated in red. The yellow, blue and green sequences corresponding to amplicons Z-1, Z23, and Z4, respectively, are used to generate GUINEA-18 and MR766^MC^ by reverse genetic approach using the ISA method. The two overlapping sequences 19 and 14 amino-acid length19 and 14 amino-acid length between the amplicons Z-1/Z-23 and Z-23/Z-4 are colored in magenta and red, respectively.
(DOCX)

S2 Fig Plaque morphology and size of infectious clones MR766^MC^ and GUINEA-18. Plaques produced by MR766^MC^ and GUINEA-18 at passage 2 after plaque forming assay on VeroE6 cells.
(TIF)

S3 Fig Antigenic reactivity of ZIKV E protein. VeroE6 cells were infected for 48h with GUINEA-18 and MR766^MC^ and then lysed with RIPA lysis buffer. Intracellular E protein was detected by immunoblot assay using anti-E mAb 4G2 on RIPA cell lysate samples. *β*-tubulin served as loading-protein control.
(TIF)

S4 Fig Infection of VeroE6 cells with ZIKV at high multiplicity of infection. VeroE6 cells were infected with MR766^MC^ and GUINEA-18 at an m.o.i of 1. *In* (**A**), virus production at various times p.i. *In* (**B**), FACS analysis was performed on ZIKV-infected cells using anti-*pan* flavivirus E mAb 4G2 and the percentage of 4G2-positive cells was determined at various times p.i.. The symbol showed at 72h p.i. indicates that massive cell death was observed with MR766^MC^.Asterisks indicate that the differences between experimental samples at each time point are statistically significant, using an unpaired *t* test (**** *p* < 0.0001, * *p* < 0.05).
(TIF)

S5 Fig Infection of HCM3 cells with ZIKV. HCM3 cells were infected for 48h with MR766^MC^ and GUINEA-18 at an m.o.i. of 10. *In* (**A**), virus production. *In* (**B**), FACS analysis was performed on ZIKV-infected cells using anti-*pan* flavivirus E mAb 4G2 and the percentage of 4G2-positive cells was determined. Asterisks indicate that the differences between experimental samples at each time point are statistically significant, using the unpaired *t* test (** *p* < 0.01; * *p* < 0.05).
(TIF)

S6 Fig ISG15 expression in A549 cells infected by ZIKV. A549 cells were infected for 48h with MR766^MC^ or GUINEA-18 or mock-infected (control) and then lysed with RIPA lysis buffer. Immunoblot assay using anti-ISG15 mAb was performed on RIPA cell lysate samples. *β*-tubulin served as loading-protein control.
(TIF)

S7 Fig Replication of a chimeric GUINEA-18 virus with the 5’ region from MR766^MC^ genome. A549 cells were infected for 48h with MR766^MC^, GUINEA-18 or chimeric GUINEA-18/MR766^5’region^ virus with the 5’ region of GUINEA-18 at an m.o.i. of 1. *In* (**A**), virus progeny production. *In* (**B**), FACS analysis was performed with anti-pan flavivirus E mAb 4G2. The percentage of 4G2-positive cells was determined. The results are the mean (± SEM) of two or three independent experiments. The values between GUINEA-18 and GUINEA-18/MR766^5’region^ were not statistically significant (n.s.), using the unpaired *t* test.
(TIF)

S8 Fig Positioning of ZIKV 3’NCR mutations. *In* (**A**), 3’NCR sequence alignment of GUINEA-18 and MR766^MC^. The lacking 3’ end of ZIKV-15555 3’NCR (Accession n° MN025403) was completed in GUINEA-18 by nucleotides 344 to 439 (in italic) from MR766^MC^ corresponding to nucleotides 10722 to 10807 of MR766-NIID genomic RNA (Accession n° LC002520). Mutations between GUINEA-18 (red) and MR766 (blue) are indicated in bold. *In* (**B**), the positions of seven mutations that differentiate the first 330 nucleotides of 3’NCR from GUINEA-18 (red) and MR766 (blue). The positioning of mutations on the predicted stem-loops (SLI and SLII), dumbell (DB) and pseudo-DB (ψDB) structures is based on the predicted structure of ZIKV 3’NCR [33].
(TIF)

S9 Fig Replication of chimeric MR766^MC^ virus with the 3’NCR of GUINEA-18. VeroE6 cells were infected for 48h with chimeric MR766/GUINEA-18^[3’NCR]^ virus or parental viruses (MR766^MC^ and GUINEA-18) at an m.o.i. of 0.1. *In* (**A**), virus progeny production. *In* (**B**), FACS analysis was performed with anti-E mAb 4G2. *In* (**C**), LDH activity was measured at 72h p.i. The results are the mean (± SEM) of two independent experiments. Asterisks indicate that the differences between experimental samples at each time point are statistically significant, using the unpaired *t* test and one-way ANOVA (**** *p* < 0.0001; *** *p* < 0.001; ** *p* < 0.01, *n*.*s*.: not significant).
(TIF)

S10 Fig ZIKV NS1 proteins. *In* (**A**), three-dimension structure prediction server Phyre^2^ was used to predict the 3D structures of the N-terminal region followed by the transmembrane helix (TM1) of ZIKV-15555 NS4B protein. The 3D viewing of the predicted structure was performed using the JSmol molecular visualization system. The clusters of residues GWLETRTKSDIAHLM (NS4B-3/17) and PASAWAIYAALTTLI (NS4B-36/50) have propensity for forming α-helical structure (helix α1) and TM1, respectively. The central disordered structure corresponding to the cluster of GRKEEGTTIGFSMDIDLRP (NS4B-18/35) residues includes the three mutations at positions 20/24/26 that differentiate ZIKV-15555 from MR766. *In* (**B**), sequence alignment of NS1 proteins from African ZIKV strains ZIKV-15555 and MR766-NIID, and epidemic Asia/America ZIKV strains BeH819015 (Accession n°KU365778), INTMI1 (Accession n°KU991811), and PRVABC-59 (Accession n°KU591215).
(TIF)

S11 Fig Expression levels of rNS4B proteins. A549 cells were transfected for 24h with plasmids expressing recombinant 2KNS4B (rNS4B) proteins from West African ZIKV strains MR776 and ZIKV-15555, and epidemic Asian/American ZIKV strain BeH819015. A plasmid expressing ZIKV-15555 rNS1 protein without the N-terminal 2K peptide [(Δ2K)-rNS4B^ZIKV-15555^] served as control. FACS analysis was performed using anti-FLAG antibody and the mean of fluorescence intensity (MFI) of transfected cells positive for FLAG-tagged rNS1 protein expression was measured as arbitrary units.
(TIF)

S12 Fig Stress granule formation in A549 cells subjected to a physiological stressor. A549 cells were transfected for 24h with a plasmid expressing eGFP-G3BP fusion protein and then incubated with 0.4M sorbitol for 1.5h (sorbitol) or mock-treated (control). Nuclei were stained with DAPI (blue). The cells were processed for confocal microscopy analysis. The blank arrow heads indicate the eGFP-positive condensates.
(TIF)
