# Supplementary material for: Replication properties of a contemporary Zika virus from West Africa
Source: PLoS Negl Trop Dis. 2024 Jul 5;18(7):e0012066. doi: 10.1371/journal.pntd.0012066 (PMC11253966; doi:10.1371/journal.pntd.0012066)
Supplement: S1 Table — Forward (F) and Reverse (R) primers. (DOCX) [file pntd.0012066.s001.docx]

S1_Table **Sequences of primers for ISA method and RT-qPCR used in this study**

| Primer | Sequence (5’ to 3’) | Target Genes |
| --- | --- | --- |
| **Z1 F** | GCGGCCGCTAGTTATTAATAGTAATC | 5’end of MR766 or GUINEA-18 amplicon n°1 |
| **Z1 R** | CCCCCAGGACTGCCATTCTCTTG | 3’end of MR766 or GUINEA-18 amplicon n°1 |
| **Z2 F** | GAGGCCACTGTGAGAGGCGCCAAGAG | 5’end of MR766 or GUINEA-18 amplicon n°23 |
| **Z3 R** | GAGAGCACTCTGAGTGTTCGTGTC | 3’end of MR766 or GUINEA-18 amplicon n°23 |
| **Z4 F** | CCTGAAGTGGAAGAGACACGAAC | 5’end of MR766 or GUINEA-18 amplicon n°4 |
| **Z4 R** | GCGCGCTAAGATACATTGATGAGTTTGG | 3’end of MR766 or GUINEA-18 amplicon n°4 |
| **NS1/2A F** | GTGGTCTCGGGAAAGAGTGTGG | 5’NS2B gene of MR766 amplicon n°23 |
| **NS1/2A R** | ACATGTCCACACTCTTTCCCGAG | 3’NS2A gene of MR766 or GUINEA-18 amplicon n°23 |
| **NS1/3 F** | TGGCCTCATAGCCtCGCTC | 5’NS4A gene of MR766 amplicon n°23 |
| **NS1/3 R** | CGGCCTCAGGCCGATAGAGC | 3’NS3 gene of GUINEA-18 amplicon n°23 |
| **ZIKV E-F** | gatgttgtcttggaacatg | Zika virus enveloppe (E) |
| **ZIKV E-R** | tcaaggtaggcttcaccttg | Zika virus enveloppe (E) |
| **RPLPO36B4 F** | AGATGCAGCAGATCCGCAT | Ribosomal Protein Lateral Stalk Subunit P0 |
| **RPLPO36B4 R** | GGATGGCCTTGCGCA | Ribosomal Protein Lateral Stalk Subunit P0 |
| **IFNβ-F** | TGTCAACATGACCAACAAGTGTCT | Interferon Beta 1 |
| **IFNβ-R** | GCAAGTTGTAGCTCATGGAAAGAG | Interferon Beta 1 |
| **BST2-F** | GCAGAGGTGGAGCGACTGAGAA | Bone Marrow Stromal Cell Antigen 2 |
| **BST2-R** | AGCAGGACGGACCTTCCAAGAT | Bone Marrow Stromal Cell Antigen 2 |
| **IFI6-F** | GCTGGTCTGCGATCCTGAATGG | Interferon Alpha Inducible Protein 6 |
| **IFI6-R** | GCTGCTGGCTACTCCTCATCCT | Interferon Alpha Inducible Protein 6 |
| **IFI27-F** | AATCGCCTCGTCCTCCATAGCA | Interferon Alpha Inducible Protein 27 |
| **IFI27-R** | CCTCGCAATGACAGCCGCAAT | Interferon Alpha Inducible Protein 27 |
| **IFIT1-F** | GCGCTGGGTATGCGATCTCT | Interferon Induced Protein with Tetratricopeptide Repeats 1 |
| **IFIT1-R** | AAGCGGACAGCCTGCCTTAG | Interferon Induced Protein with Tetratricopeptide Repeats 1 |
| **IFITM1-F** | TCCTTCCAAGGTCCACCGTGAT | Interferon Induced Transmembrane Protein 1 |
| **IFITM1-R** | CGTCGCCAACCATCTTCCTGTC | Interferon Induced Transmembrane Protein 1 |
| **ISG15-F** | TGGCGGGCAACGAATT | Interferon-Stimulated Gene 15 |
| **ISG15-R** | GGGTGATCTGCGCCTTCA | Interferon-Stimulated Gene 15 |
| **MX1-F** | CACCAGCGACAAGCGGAAGTT | MX Dynamin Like GTPase 1 |
| **MX1-R** | AGTCGTCAGTCCAGTGGCTACC | MX Dynamin Like GTPase 1 |
| **MX2-F** | GAACAATCAGCCACCACCAGGA | MX Dynamin Like GTPase 2 |
| **MX2-R** | TTCAGCACCAGCGGACACCT | MX Dynamin Like GTPase 2 |
| **OAS1-F** | GCAGACGATGAGACCGACGAT | 2'-5'-Oligoadenylate Synthetase 1 |
| **OAS1-R** | GCACTGGCATTCAGAGGATGGT | 2'-5'-Oligoadenylate Synthetase 1 |
| **OAS2-F** | TGCTCTCGGTGCTTCCAACTCA | 2'-5'-Oligoadenylate Synthetase 2 |
| **OAS2-R** | TGGCTGCTGGCATAGAGGATGT | 2'-5'-Oligoadenylate Synthetase 2 |
| **OAS3-F** | ATGCCGACCTCGTGGTGTTC | 2'-5'-Oligoadenylate Synthetase 3 |
| **OAS3-R** | AACTGCCGCTCCTGTTGACAT | 2'-5'-Oligoadenylate Synthetase 3 |
| **USP18-F** | CCATCGTGCCTGGCTCACAT | Ubiquitin Specific Peptidase 18 |
| **USP18-R** | AACCAACCAGGCCATGAGGG | Ubiquitin Specific Peptidase 18 |
| **VP-F** | CGTGAGCATCGTGAGCAATG | Viperin |
| **VP-R** | GCTGTCACAGGAGATAGCGA | Viperin |
| **XAF1-F** | GCCTACTTGCTGTGGTGGTCTT | XIAP Associated Factor 1 |
| **XAF1-R** | ATGTTCCTTCGACGCCTGGTT | XIAP Associated Factor 1 |
