# Supplementary material for: Replication properties of a contemporary Zika virus from West Africa
Source: PLoS Negl Trop Dis. 2024 Jul 5;18(7):e0012066. doi: 10.1371/journal.pntd.0012066 (PMC11253966; doi:10.1371/journal.pntd.0012066)
Supplement: S1 Fig — Alignment of polyproteins (amino-acids 1 to 3423) from viral strains ZIKV-15555 (Accession n° MN025403) and MR766-NIID (Accession n° LC002520). Arrows indicate the starting amino acid of each mature viral protein. The amino-acid substitutions between ZIKV-15555 and MR766-NIID - are indicated in red. The yellow, blue and green sequences corresponding to amplicons Z-1, Z23, and Z4, respectively, are used to generate GUINEA-18 and MR766MC by reverse genetic approach using the ISA method. The two overlapping sequences 19 and 14 amino-acid length19 and 14 amino-acid length between the amplicons Z-1/Z-23 and Z-23/Z-4 are colored in magenta and red, respectively. (DOCX) [file pntd.0012066.s002.docx]

-> C

10 20 30 40 50 60

Guinea MKNPK**R**KSGGFRIVNMLKRGVARVNPLGGLKRLPAGLLLGHGPIRMVLAILAFLRFTAIK **ZIKV-15555**

:::::.::::::::::::::::::::::::::::::::::::::::::::::::::::::

MR766 MKNPK**K**KSGGFRIVNMLKRGVARVNPLGGLKRLPAGLLLGHGPIRMVLAILAFLRFTAIK **MR766-NIID**

70 80 90 100 110 120

Guinea PSLGLINRWGSVGKKEAMEIIKKFKKDLAAMLRIINARKERKRRGADTSIGI**V**GLLLTTA **ZIKV-15555**

::::::::::::::::::::::::::::::::::::::::::::::::::::.:::::::

MR766 PSLGLINRWGSVGKKEAMEIIKKFKKDLAAMLRIINARKERKRRGADTSIGI**I**GLLLTTA **MR766-NIID**

->prM/M

130 140 150 160 170 180

Guinea MAAEITRRGSAYYMYLDRSDAGKAISFATTLGVNKCHVQIMDLGHMCDATMSYECPMLDE **ZIKV-15555**

::::::::::::::::::::::::::::::::::::::::::::::::::::::::::::

MR766 MAAEITRRGSAYYMYLDRSDAGKAISFATTLGVNKCHVQIMDLGHMCDATMSYECPMLDE **MR766-NIID**

190 200 210 220 230 240

Guinea GVEPDDVDCWCNTTSTWVVYGTCHHKKGEARRSRRAVTLPSHSTRKLQTRSQTWLESREY **ZIKV-15555**

::::::::::::::::::::::::::::::::::::::::::::::::::::::::::::

MR766 GVEPDDVDCWCNTTSTWVVYGTCHHKKGEARRSRRAVTLPSHSTRKLQTRSQTWLESREY **MR766-NIID**

->E

250 260 270 280 290 300

Guinea TKHLIKVENWIFRNPGFALVAVAIAWLLGSSTSQKVIYLVMILLIAPAYSIRCIGVSNRD **ZIKV-15555**

::::::::::::::::::::::::::::::::::::::::::::::::::::::::::::

MR766 TKHLIKVENWIFRNPGFALVAVAIAWLLGSSTSQKVIYLVMILLIAPAYSIRCIGVSNRD **MR766-NIID**

310 320 330 340 350 360

Guinea FVEGMSGGTWVDVVLEHGGCVTVMAQDKPTVDIELVTTTVSNMAEVRSYCYEASISDMAS **ZIKV-15555**

::::::::::::::::::::::::::::::::::::::::::::::::::::::::::::

MR766 FVEGMSGGTWVDVVLEHGGCVTVMAQDKPTVDIELVTTTVSNMAEVRSYCYEASISDMAS **MR766-NIID**

370 380 390 400 410 420

Guinea DSRCPTQGEAYLDKQSDTQYVCKRTLVDRGWGNGCGLFGKGSLVTCAKFTCSKKMTGKSI **ZIKV-15555**

::::::::::::::::::::::::::::::::::::::::::::::::::::::::::::

MR766 DSRCPTQGEAYLDKQSDTQYVCKRTLVDRGWGNGCGLFGKGSLVTCAKFTCSKKMTGKSI **MR766-NIID**

430 440 450 460 470 480

Guinea QPENLEYRIMLSVHGSQHSGM**I**VND**T**G**H**ETDENRAKVEVTPNSPRAEATLGGFGSLGLDC **ZIKV-15555**

::::::::::::::::::::: ::: :.::::::::::::::::::::::::::::::::

MR766 QPENLEYRIMLSVHGSQHSGM**T**VND**I**G**Y**ETDENRAKVEVTPNSPRAEATLGGFGSLGLDC **MR766-NIID**

490 500 510 520 530 540

Guinea EPRTGLDFSDLYYLTMNNKHWLVHKEWFHDIPLPWHAGADTGTPHWNNKEALVEFKDAHA **ZIKV-15555**

::::::::::::::::::::::::::::::::::::::::::::::::::::::::::::

MR766 EPRTGLDFSDLYYLTMNNKHWLVHKEWFHDIPLPWHAGADTGTPHWNNKEALVEFKDAHA **MR766-NIID**

550 560 570 580 590 600

Guinea KRQTVVVLGSQEGAVHTALAGALEAEMDGAKG**R**LFSGHLKCRLKMDKLRLKGVSYSLCTA **ZIKV-15555**

::::::::::::::::::::::::::::::::.:::::::::::::::::::::::::::

MR766 KRQTVVVLGSQEGAVHTALAGALEAEMDGAKG**K**LFSGHLKCRLKMDKLRLKGVSYSLCTA **MR766-NIID**

610 620 630 640 650 660

Guinea AFTFTKVPAETLHGTVTVEVQYAGTDGPCK**V**P**A**QMAVDMQTLTPVGRLITANPVITESTE **ZIKV-15555**

::::::::::::::::::::::::::::::.:.:::::::::::::::::::::::::::

MR766 AFTFTKVPAETLHGTVTVEVQYAGTDGPCK**I**P**V**QMAVDMQTLTPVGRLITANPVITESTE **MR766-NIID**

670 680 690 700 710 720

Guinea NSKMMLELDPPFGDSYIVIGVGDKKITHHWHRSGSTIGKAFEATVRGAKRMAVLGDTAWD **ZIKV-15555**

:::::::::::::::::::::::::::::::::::::::::...................................................................................................................................................

MR766 NSKMMLELDPPFGDSYIVIGVGDKKITHHWHRSGSTIGKAFEATVRGAKRMAVLGDTAWD **MR766-NIID**

730 740 750 760 770 780

Guinea FGSVGGVFNSLGKGIHQIFGAAFKSLFGGMSWFSQILIGTLLVWLGLNTKNGSISLTCLA **ZIKV-15555**

:::::::::::::::::::::::::::::::::::::::::...............................................................................................................................................

MR766 FGSVGGVFNSLGKGIHQIFGAAFKSLFGGMSWFSQILIGTLLVWLGLNTKNGSISLTCLA **MR766-NIID**

-> NS1

790 800 810 820 830 840

LGGVMIFLSTAVSADVGCSVDFSKKETRCGTGVF**V**YNDVEAWRDRYKYHPDSPRRLAAAV **ZIKV-15555**

:::::::::::::::::::::::::::::::::::::::::...............................................................................................................................................

MR766 LGGVMIFLSTAVSADVGCSVDFSKKETRCGTGVF**I**YNDVEAWRDRYKYHPDSPRRLAAAV **MR766-NIID**

850 860 870 880 890 900

Guinea KQAWEEGICGISSVSRMENIMWKSVEGELNAILEENGVQLTVVVG**P**VKNPMWRGPQRLPV **ZIKV-15555**

::::::::::::::::::::::::::::::::::::::::::::: ::::::::::::::

MR766 KQAWEEGICGISSVSRMENIMWKSVEGELNAILEENGVQLTVVVG**S**VKNPMWRGPQRLPV **MR766-NIID**

910 920 930 940 950 960

Guinea PVNELPHGWKAWGKSYFVRAAKTNNSFVVDGDTLKECPL**K**HRAWNSFLVEDHGFG**I**FHTS **ZIKV-15555**

:::::::::::::::::::::::::::::::::::::::.:::::::::::::::.::::

MR766 PVNELPHGWKAWGKSYFVRAAKTNNSFVVDGDTLKECPL**E**HRAWNSFLVEDHGFG**V**FHTS **MR766-NIID**

970 980 990 1000 1010 1020

Guinea VWLKVREDYSLECDPAVIGTAVKG**K**EAAHSDLGYWIESEKNDTWRL**R**RAHLIEMKTCEWP **ZIKV-15555**

::::::::::::::::::::::::.:::::::::::::::::::::.:::::::::::::

MR766 VWLKVREDYSLECDPAVIGTAVKG**R**EAAHSDLGYWIESEKNDTWRL**K**RAHLIEMKTCEWP **MR766-NIID**

1030 1040 1050 1060 1070 1080

Guinea KSHTLWTDGVEESDLIIPKSLAGPLSHHNTREGYRTQVKGPWHSEELEIRFEECPGTKV**H** **ZIKV-15555**

:::::::::::::::::::::::::::::::::::::::::::::::::::::::::::.

MR766 KSHTLWTDGVEESDLIIPKSLAGPLSHHNTREGYRTQVKGPWHSEELEIRFEECPGTKV**Y** **MR766-NIID**

1090 1100 1110 1120 1130 1140

Guinea VEETCGTRGPSLRSTTASGRVIEEWCCRECTMPPLSFRAKDGCWYGMEIRPRKEPESNLV **ZIKV-15555**

::::::::::::::::::::::::::::::::::::::::::::::::::::::::::::

MR766 VEETCGTRGPSLRSTTASGRVIEEWCCRECTMPPLSFRAKDGCWYGMEIRPRKEPESNLV **MR766-NIID**

-> NS2A

1150 1160 1170 1180 1190 1200

Guinea RSMVTAGSTDHMDHFSLGVLVILLMVQEGLKKRMTTKIIMSTSMAVLV**A**M**V**LGGFSMSDL **ZIKV-15555**

::::::::::::::::::::::::::::::::::::::::::::::::.:.:::::::::

MR766 RSMVTAGSTDHMDHFSLGVLVILLMVQEGLKKRMTTKIIMSTSMAVLV**V**M**I**LGGFSMSDL **MR766-NIID**

1210 1220 1230 1240 1250 1260

Guinea AKLVILMGATFAEMNTGGDVAHLALVAAFKVRPALLVSFIFRANWTPRESMLLALASCLL **ZIKV-15555**

::::::::::::::::::::::::::::::::::::::::::::::::::::::::::::

MR766 AKLVILMGATFAEMNTGGDVAHLALVAAFKVRPALLVSFIFRANWTPRESMLLALASCLL **MR766-NIID**

1270 1280 1290 1300 1310 1320

Guinea QTAISALEG**E**LMVL**V**NGFALAWLAIRAMAVPRTDNIAL**A**ILAALTPLARGTLLVAWRAGL **ZIKV-15555**

:::::::::.::::.::::::::::::::::::::::: :::::::::::::::::::::

MR766 QTAISALEG**D**LMVL**I**NGFALAWLAIRAMAVPRTDNIAL**P**ILAALTPLARGTLLVAWRAGL **MR766-NIID**

-> NS2B

1330 1340 1350 1360 1370 1380

Guinea ATCGG**F**MLLSLKGKGSVKKNLPFVMALGLTAVR**M**VDPINVVGLLLLTRSGKRSWPPSEVL **ZIKV-15555**

:::::.:::::::::::::::::::::::::::.::::::::::::::::::::::::::

MR766 ATCGG**I**MLLSLKGKGSVKKNLPFVMALGLTAVR**V**VDPINVVGLLLLTRSGKRSWPPSEVL **MR766-NIID**

1390 1400 1410 1420 1430 1440

Guinea TAVGLICALAGGFAKADIEMAGPMAAVGLLIVSYVVSGKSVDMYIERAGDITWEKDAEVT **ZIKV-15555**

::::::::::::::::::::::::::::::::::::::::::::::::::::::::::::

MR766 TAVGLICALAGGFAKADIEMAGPMAAVGLLIVSYVVSGKSVDMYIERAGDITWEKDAEVT **MR766-NIID**

1450 1460 1470 1480 1490 1500

Guinea GNSPRLDVALDESGDFSLVEEDGPPMREIILKVVLMAICGMNPIAIPFAAGAWYVYVKTG **ZIKV-15555**

::::::::::::::::::::::::::::::::::::::::::::::::::::::::::::

MR766 GNSPRLDVALDESGDFSLVEEDGPPMREIILKVVLMAICGMNPIAIPFAAGAWYVYVKTG **MR766-NIID**

-> NS3

1510 1520 1530 1540 1550 1560

Guinea KRSGALWDVPAPKEVKKGETTDGVYRVMTRRLLGSTQVGVGVMQEGVFHTMWHVTKGAAL **ZIKV-15555**

::::::::::::::::::::::::::::::::::::::::::::::::::::::::::::

MR766 KRSGALWDVPAPKEVKKGETTDGVYRVMTRRLLGSTQVGVGVMQEGVFHTMWHVTKGAAL **MR766-NIID**

1570 1580 1590 1600 1610 1620

Guinea RSGEGRLDPYWGDVKQDLVSYCGPWKLDAAWDGLSEVQLLAVPPGERARNIQTLPGIFKT **ZIKV-15555**

::::::::::::::::::::::::::::::::::::::::::::::::::::::::::::

MR766 RSGEGRLDPYWGDVKQDLVSYCGPWKLDAAWDGLSEVQLLAVPPGERARNIQTLPGIFKT **MR766-NIID**

1630 1640 1650 1660 1670 1680

Guinea KDGDIGAVALDYPAGTSGSPILDKCGRVIGLYGNGVV**V**KNGSYVSAITQGKREEE**A**PVEC **ZIKV-15555**

:::::::::::::::::::::::::::::::::::::.:::::::::::::::::.::::

MR766 KDGDIGAVALDYPAGTSGSPILDKCGRVIGLYGNGVV**I**KNGSYVSAITQGKREEE**T**PVEC **MR766-NIID**

1690 1700 1710 1720 1730 1740

Guinea FEPSML**R**KKQLTVLDLHPGAGKTRRVLPEIVREAIKKRLRT**A**ILAPTRVVAAEMEEALRG **ZIKV-15555**

::::::.::::::::::::::::::::::::::::::::::.::::::::::::::::::

MR766 FEPSML**K**KKQLTVLDLHPGAGKTRRVLPEIVREAIKKRLRT**V**ILAPTRVVAAEMEEALRG **MR766-NIID**

1750 1760 1770 1780 1790 1800

Guinea LPVRYMTTAVNV**I**HSGTEIVDLMCHATFTSRLLQPIRVPNYNLYIMDEAHFTDPSSIAAR **ZIKV-15555**

:::::::::::: :::::::::::::::::::::::::::::::::::::::::::::::

MR766 LPVRYMTTAVNV**T**HSGTEIVDLMCHATFTSRLLQPIRVPNYNLYIMDEAHFTDPSSIAAR **MR766-NIID**

1810 1820 1830 1840 1850 1860

Guinea GYISTRVEMGEAAAIFMTATPPGTRDAFPDSNSPIMDTEVEVPERAWSSGFDWVTDHSG**R** **ZIKV-15555**

:::::::::::::::::::::::::::::::::::::::::::::::::::::::::::.

MR766 GYISTRVEMGEAAAIFMTATPPGTRDAFPDSNSPIMDTEVEVPERAWSSGFDWVTDHSG**K** **MR766-NIID**

1870 1880 1890 1900 1910 1920

Guinea T**I**WFVPSVRNGNEIAACLTKAGKRVIQLSRKTFETEFQKTKNQEWDFVITTDISEMGANF **ZIKV-15555**

:.::::::::::::::::::::::::::::::::::::::::::::::::::::::::::

MR766 T**V**WFVPSVRNGNEIAACLTKAGKRVIQLSRKTFETEFQKTKNQEWDFVITTDISEMGANF **MR766-NIID**

1930 1940 1950 1960 1970 1980

Guinea KADRVIDSRRCLKPVILDGERVILAGPMPVTHASAAQRRGRIGRNPNKPGDEYMYGGGCA **ZIKV-15555**

::::::::::::::::::::::::::::::::::::::::::::::::::::::::::::

MR766 KADRVIDSRRCLKPVILDGERVILAGPMPVTHASAAQRRGRIGRNPNKPGDEYMYGGGCA **MR766-NIID**

1990 2000 2010 2020 2030 2040

Guinea ETDE**D**HAHWLEARMLLDNIYLQDGLIASLYRPEADKVAAIEGEFKLRTEQRKTFVELMKR **ZIKV-15555**

:::: :::::::::::::::::::::::::::::::::::::::::::::::::::::::

MR766 ETDE**G**HAHWLEARMLLDNIYLQDGLIASLYRPEADKVAAIEGEFKLRTEQRKTFVELMKR **MR766-NIID**

2050 2060 2070 2080 2090 2100

Guinea GDLPVWLAYQVASAGITYTDRRWCFDGTTNNTIMEDSVPAEVWTKYGEKRVLKPRWMDAR **ZIKV-15555**

::::::::::::::::::::::::::::::::::::::::::::::::::::::::::::

MR766 GDLPVWLAYQVASAGITYTDRRWCFDGTTNNTIMEDSVPAEVWTKYGEKRVLKPRWMDAR **MR766-NIID**

-> NS4A

2110 2120 2130 2140 2150 2160

Guinea VCSDHAALKSFKEFAAGKRGAALGVM**D**ALGTLPGHMTERFQEAIDNLAVLMRAETGSRPY **ZIKV-15555**

::::::::::::::::::::::::::.:::::::::::::::::::::::::::::::::

MR766 VCSDHAALKSFKEFAAGKRGAALGVM**E**ALGTLPGHMTERFQEAIDNLAVLMRAETGSRPY **MR766-NIID**

2170 2180 2190 2200 2210 2220

Guinea KAAAAQLPETLETIMLLGLLGTVSLGIFFVLMRNKGIGKMGFGMVTLGASAWLMWLSEIE **ZIKV-15555**

::::::::::::::::::::::::::::::::::::::::::::::::::::::::::::

MR766 KAAAAQLPETLETIMLLGLLGTVSLGIFFVLMRNKGIGKMGFGMVTLGASAWLMWLSEIE **MR766-NIID**

-> NS4B

2230 2240 2250 2260 2270 2280

Guinea PARIACVLIVVFLLLVVLIPEPEKQRSPQDNQMAIIIMVAVGLLGLITANELGWLERTK**S** **ZIKV-15555**

:::::::::::::::::::::::::::::::::::::::::::::::::::::::::::.

MR766 PARIACVLIVVFLLLVVLIPEPEKQRSPQDNQMAIIIMVAVGLLGLITANELGWLERTK**N** **MR766-NIID**

2290 2300 2310 2320 2330 2340

Guinea DIAHLMGR**K**EEG**T**T**I**GFSMDIDLRPASAWAIYAALTTLITPAVQHAVTTSYNNYSLMAMA **ZIKV-15555**

::::::::.:::.:.:::::::::::::::::::::::::::::::::::::::::::::

MR766 DIAHLMGR**R**EEG**A**T**M**GFSMDIDLRPASAWAIYAALTTLITPAVQHAVTTSYNNYSLMAMA **MR766-NIID**

2350 2360 2370 2380 2390 2400

Guinea TQAGVLFGMGKGMPFYAWD**F**GVPLLM**I**GCYSQLTPLTLIVAIILLVAHYMYLIPGLQAAA **ZIKV-15555**

:::::::::::::::::::.::::::.:::::::::::::::::::::::::::::::::

MR766 TQAGVLFGMGKGMPFYAWD**L**GVPLLM**M**GCYSQLTPLTLIVAIILLVAHYMYLIPGLQAAA **MR766-NIID**

2410 2420 2430 2440 2450 2460

Guinea ARAAQKRTAAGIMKNPVVDGIVVTDIDTMTIDPQVEKKMGQVLLIAVA**V**SSAVLLRTAWG **ZIKV-15555**

::::::::::::::::::::::::::::::::::::::::::::::::.:::::::::::

MR766 ARAAQKRTAAGIMKNPVVDGIVVTDIDTMTIDPQVEKKMGQVLLIAVA**I**SSAVLLRTAWG **MR766-NIID**

-> NS5

2470 2480 2490 2500 2510 2520

Guinea WGEAGALITAATSTLWEGSPNKYWNSSTATSLCNIFRGSYLAGASLIYTVTRNAGLVKRR **ZIKV-15555**

::::::::::::::::::::::::::::::::::::::::::::::::::::::::::::

MR766 WGEAGALITAATSTLWEGSPNKYWNSSTATSLCNIFRGSYLAGASLIYTVTRNAGLVKRR **MR766-NIID**

2530 2540 2550 2560 2570 2580

Guinea GGGTGETLGEKWKARLNQMSALEFYSYKKSGITEVCREEARRALKDGVATGGHAVSRGSA **ZIKV-15555**

::::::::::::::::::::::::::::::::::::::::::::::::::::::::::::

MR766 GGGTGETLGEKWKARLNQMSALEFYSYKKSGITEVCREEARRALKDGVATGGHAVSRGSA **MR766-NIID**

2590 2600 2610 2620 2630 2640

Guinea KLRWLVERGYLQP**H**GKVVDLGCGRGGWSYYAATIRKVQEVRGYTKGGPGHEEPMLVQSYG **ZIKV-15555**

:::::::::::::.::::::::::::::::::::::::::::::::::::::::::::::

MR766 KLRWLVERGYLQP**Y**GKVVDLGCGRGGWSYYAATIRKVQEVRGYTKGGPGHEEPMLVQSYG **MR766-NIID**

2650 2660 2670 2680 2690 2700

Guinea WNIVRLKSGVDVFHMAAEPCDTLLCDIGESSSSPEVEETRTLRVLSMVGDWLEKRPGAFC **ZIKV-15555**

::::::::::::::::::::::::::::::::::::::::::::::::::::::::::::

MR766 WNIVRLKSGVDVFHMAAEPCDTLLCDIGESSSSPEVEETRTLRVLSMVGDWLEKRPGAFC **MR766-NIID**

2710 2720 2730 2740 2750 2760

Guinea IKVLCPYTSTMMETMERLQRRHGGGLVRVPLSRNSTHEMYWVSGAKSNIIKSVSTTSQLL **ZIKV-15555**

::::::::::::::::::::::::::::::::::::::::::::::::::::::::::::

MR766 IKVLCPYTSTMMETMERLQRRHGGGLVRVPLSRNSTHEMYWVSGAKSNIIKSVSTTSQLL **MR766-NIID**

2770 2780 2790 2800 2810 2820

Guinea LGRMDGPRRPVKYEEDVNLGSGTRAVASCAEAPNMKIIGRRIERIRNEHAETWF**F**DENHP **ZIKV-15555**

::::::::::::::::::::::::::::::::::::::::::::::::::::::.:::::

MR766 LGRMDGPRRPVKYEEDVNLGSGTRAVASCAEAPNMKIIGRRIERIRNEHAETWF**L**DENHP **MR766-NIID**

2830 2840 2850 2860 2870 2880

Guinea YRTWAYHGSYEAPTQGSASSLVNGVVRLLSKPWDVVTGVTGIAMTDTTPYGQQRVFKEKV **ZIKV-15555**

::::::::::::::::::::::::::::::::::::::::::::::::::::::::::::

MR766 YRTWAYHGSYEAPTQGSASSLVNGVVRLLSKPWDVVTGVTGIAMTDTTPYGQQRVFKEKV **MR766-NIID**

2890 2900 2910 2920 2930 2940

Guinea DTRVPDPQEGTRQVMN**M**VSSWLWKELGKRKRPRVCTKEEFINKVRSNAALGAIF**V**EEKEW **ZIKV-15555**

::::::::::::::::.::::::::::::::::::::::::::::::::::::: :::::

MR766 DTRVPDPQEGTRQVMN**I**VSSWLWKELGKRKRPRVCTKEEFINKVRSNAALGAIF**E**EEKEW **MR766-NIID**

2950 2960 2970 2980 2990 3000

Guinea KTAVEAVNDPRFWALVD**K**EREHHLRGECHSCVYNMMGKREKKQGEFGKAKGSRAIWYMWL **ZIKV-15555**

:::::::::::::::::.::::::::::::::::::::::::::::::::::::::::::

MR766 KTAVEAVNDPRFWALVD**R**EREHHLRGECHSCVYNMMGKREKKQGEFGKAKGSRAIWYMWL **MR766-NIID**

3010 3020 3030 3040 3050 3060

Guinea GARFLEFEALGFLNEDHWMGRENSGGGVEGLGLQRLGYILEEMNRAPGG**R**MYADDTAGWD **ZIKV-15555**

:::::::::::::::::::::::::::::::::::::::::::::::::.::::::::::

MR766 GARFLEFEALGFLNEDHWMGRENSGGGVEGLGLQRLGYILEEMNRAPGG**K**MYADDTAGWD **MR766-NIID**

3070 3080 3090 3100 3110 3120

Guinea TRISKFDLENEALITNQMEEGHR**A**LALAVIKYTYQNKVVKVLRPAEGGKTVMDIISRQDQ **ZIKV-15555**

:::::::::::::::::::::::.::::::::::::::::::::::::::::::::::::

MR766 TRISKFDLENEALITNQMEEGHR**T**LALAVIKYTYQNKVVKVLRPAEGGKTVMDIISRQDQ **MR766-NIID**

3130 3140 3150 3160 3170 3180

Guinea RGSGQVVTYALNTFTNLVVQLIRNMEAEEVLEM**H**DLWLLRKPEKVTRWLQS**D**GWDRLKRM **ZIKV-15555**

:::::::::::::::::::::::::::::::::.:::::::::::::::::.::::::::

MR766 RGSGQVVTYALNTFTNLVVQLIRNMEAEEVLEM**Q**DLWLLRKPEKVTRWLQS**N**GWDRLKRM **MR766-NIID**

3190 3200 3210 3220 3230 3240

Guinea AVSGDDCVVKPIDDRFAHALRFLNDMGKVRKDTQEWKPSTGWSNWEEVPFCSHHFNKL**H**L **ZIKV-15555**

::::::::::::::::::::::::::::::::::::::::::::::::::::::::::.:

MR766 AVSGDDCVVKPIDDRFAHALRFLNDMGKVRKDTQEWKPSTGWSNWEEVPFCSHHFNKL**Y**L **MR766-NIID**

3250 3260 3270 3280 3290 3300

Guinea KDGRSIVVPCRHQDELIGRARVSPGAGWSIRETACLAKSYAQMWQLLYFHRRDLRLMANA **ZIKV-15555**

::::::::::::::::::::::::::::::::::::::::::::::::::::::::::::

MR766 KDGRSIVVPCRHQDELIGRARVSPGAGWSIRETACLAKSYAQMWQLLYFHRRDLRLMANA **MR766-NIID**

3310 3320 3330 3340 3350 3360

Guinea ICSAVPVDWVPTGRTTWSIHGKGEWMTTEDMLMVWNRVWIEENDHMEDKTPV**K**KWTDIPY **ZIKV-15555**

:::::::::::::::::::::::::::::::::::::::::::::::::::: :::::::

MR766 ICSAVPVDWVPTGRTTWSIHGKGEWMTTEDMLMVWNRVWIEENDHMEDKTPV**T**KWTDIPY **MR766-NIID**

3370 3380 3390 3400 3410 3420

Guinea LGKREDLWCGSLIGHRPRTTWAENIKDTVNMVRRIIGDEEKYMDYLSTQVRYLGEEGSTP **ZIKV-15555**

::::::::::::::::::::::::::::::::::::::::::::::::::::::::::::

MR766 LGKREDLWCGSLIGHRPRTTWAENIKDTVNMVRRIIGDEEKYMDYLSTQVRYLGEEGSTP **MR766-NIID**

3423

Guinea GVL **ZIKV-15555**

:::

MR766 GVL **MR766-NIID**
