# Supplementary figures and images for: Replication properties of a contemporary Zika virus from West Africa
Source: PLoS Negl Trop Dis. 2024 Jul 5;18(7):e0012066. doi: 10.1371/journal.pntd.0012066 (PMC11253966; doi:10.1371/journal.pntd.0012066)

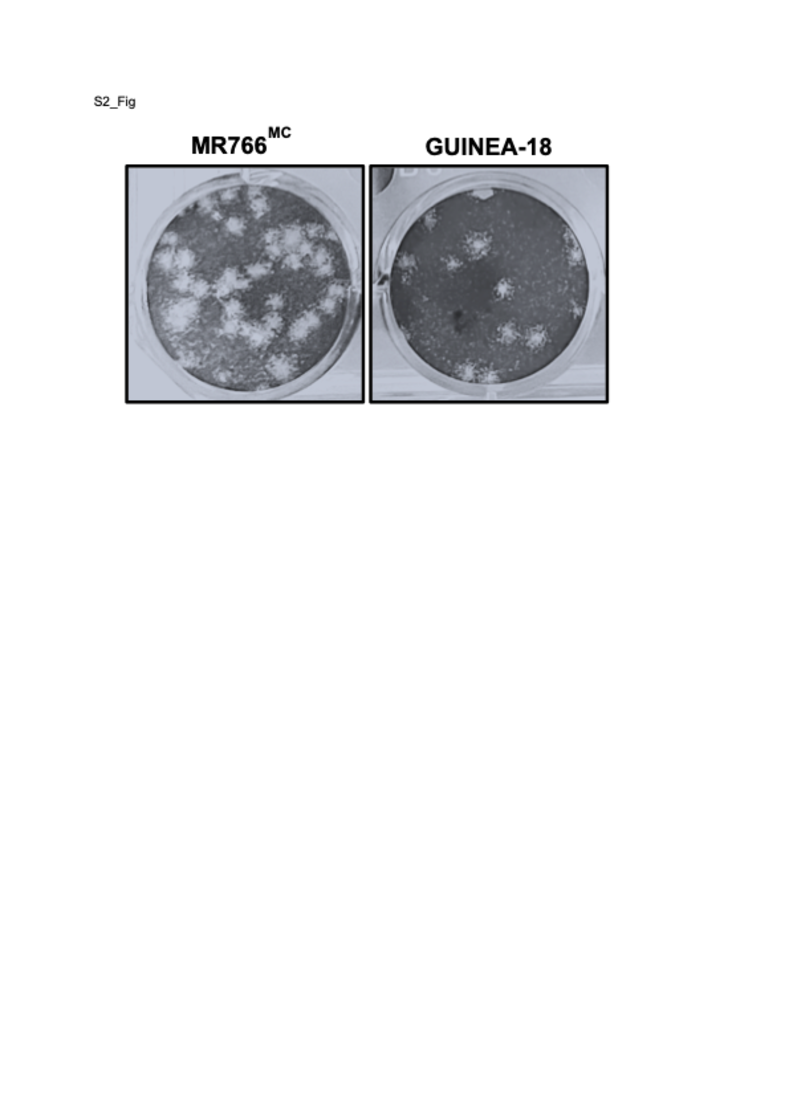

Supplement: S2 Fig — Plaques produced by MR766MC and GUINEA-18 at passage 2 after plaque forming assay on VeroE6 cells. (TIF) [file pntd.0012066.s003.tif]

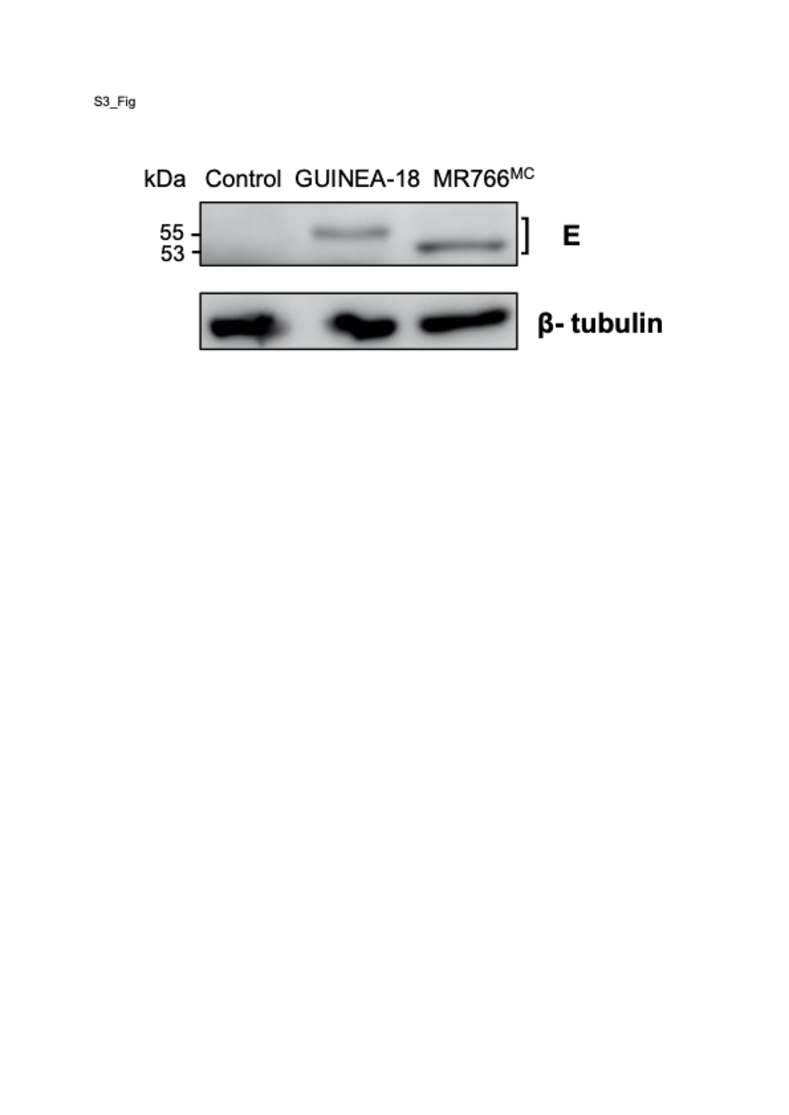

Supplement: S3 Fig — VeroE6 cells were infected for 48h with GUINEA-18 and MR766MC and then lysed with RIPA lysis buffer. Intracellular E protein was detected by immunoblot assay using anti-E mAb 4G2 on RIPA cell lysate samples. β-tubulin served as loading-protein control. (TIF) [file pntd.0012066.s004.tif]

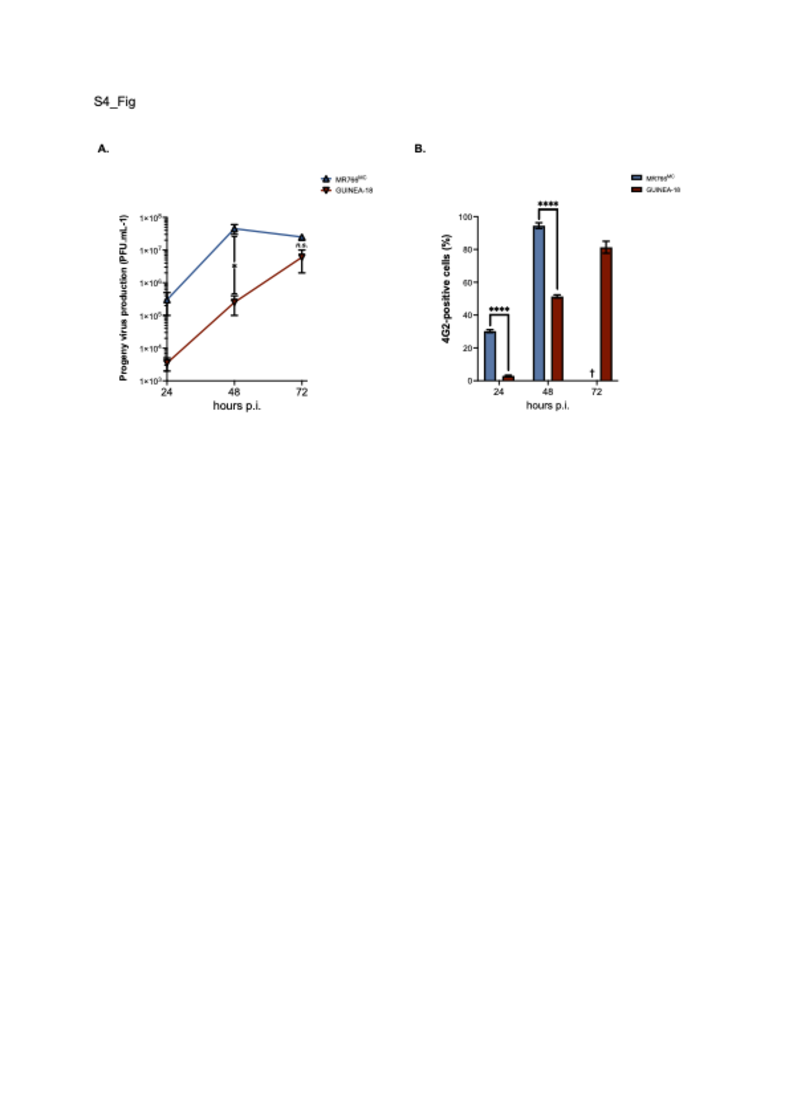

Supplement: S4 Fig — VeroE6 cells were infected with MR766MC and GUINEA-18 at an m.o.i of 1. In (A), virus production at various times p.i. In (B), FACS analysis was performed on ZIKV-infected cells using anti-pan flavivirus E mAb 4G2 and the percentage of 4G2-positive cells was determined at various times p.i.. The symbol showed at 72h p.i. indicates that massive cell death was observed with MR766MC.Asterisks indicate that the differences between experimental samples at each time point are statistically significant, using an unpaired t test (**** p < 0.0001, * p < 0.05). (TIF) [file pntd.0012066.s005.tif]

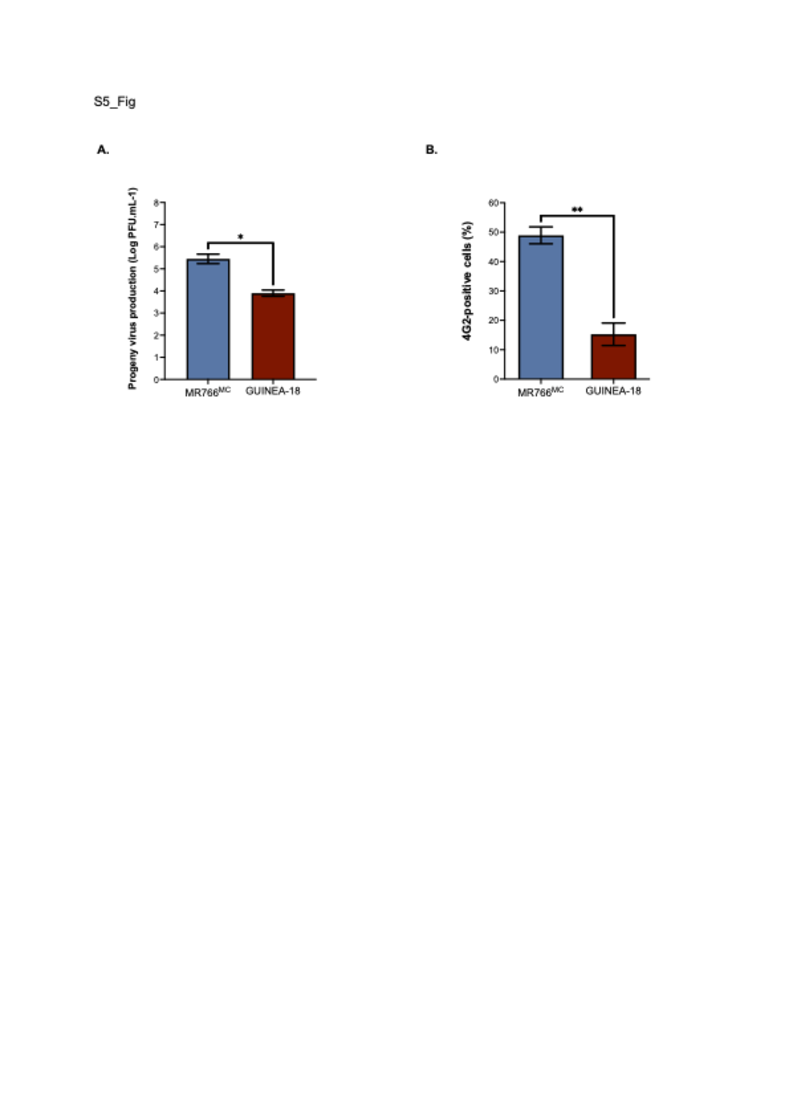

Supplement: S5 Fig — HCM3 cells were infected for 48h with MR766MC and GUINEA-18 at an m.o.i. of 10. In (A), virus production. In (B), FACS analysis was performed on ZIKV-infected cells using anti-pan flavivirus E mAb 4G2 and the percentage of 4G2-positive cells was determined. Asterisks indicate that the differences between experimental samples at each time point are statistically significant, using the unpaired t test (** p < 0.01; * p < 0.05). (TIF) [file pntd.0012066.s006.tif]

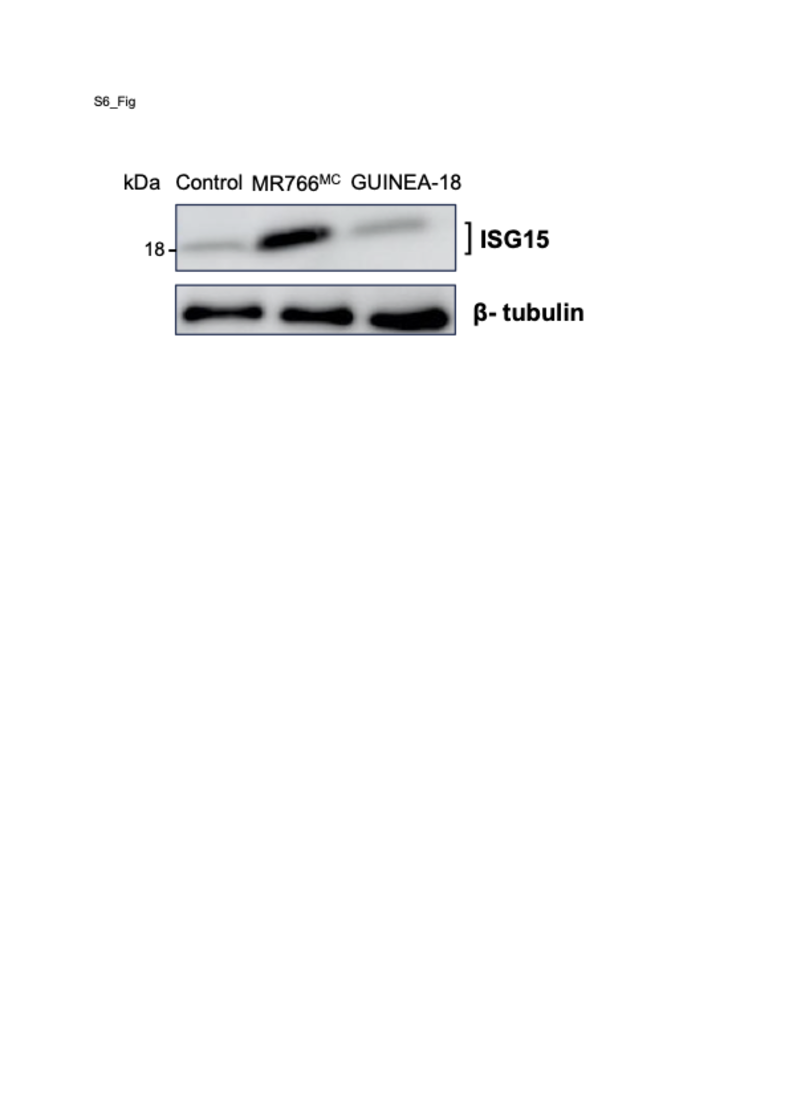

Supplement: S6 Fig — A549 cells were infected for 48h with MR766MC or GUINEA-18 or mock-infected (control) and then lysed with RIPA lysis buffer. Immunoblot assay using anti-ISG15 mAb was performed on RIPA cell lysate samples. β-tubulin served as loading-protein control. (TIF) [file pntd.0012066.s007.tif]

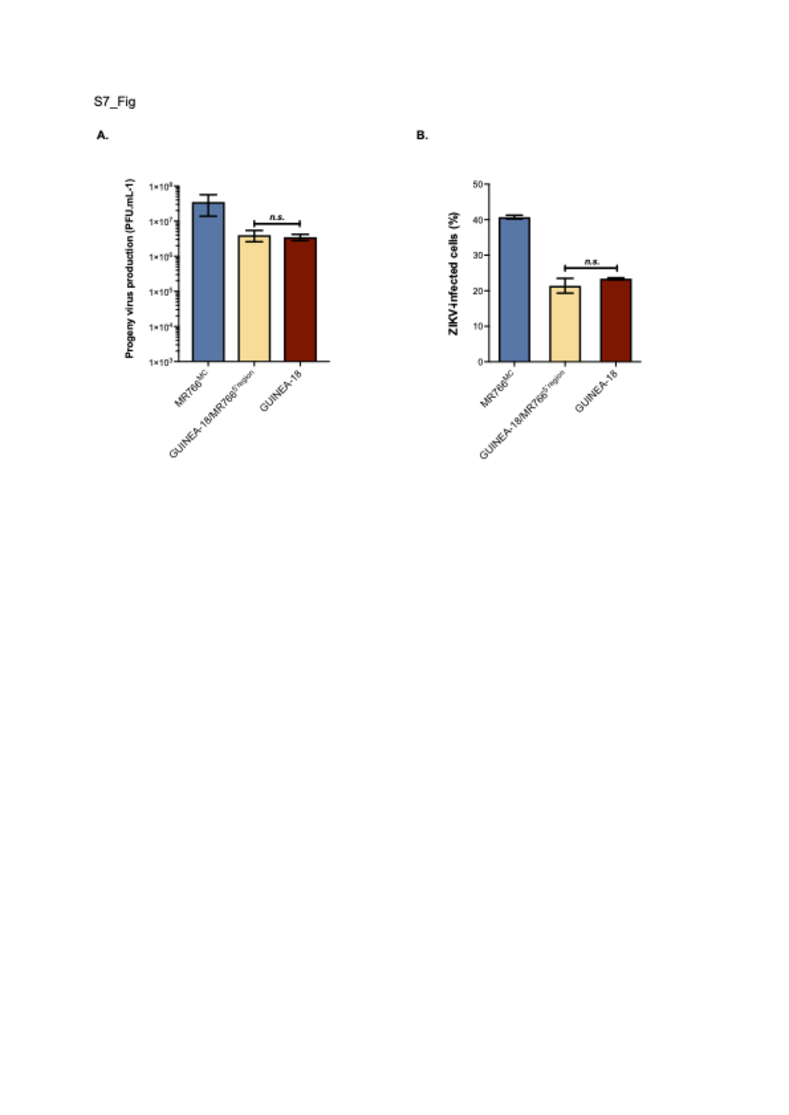

Supplement: S7 Fig — A549 cells were infected for 48h with MR766MC, GUINEA-18 or chimeric GUINEA-18/MR7665’region virus with the 5’ region of GUINEA-18 at an m.o.i. of 1. In (A), virus progeny production. In (B), FACS analysis was performed with anti-pan flavivirus E mAb 4G2. The percentage of 4G2-positive cells was determined. The results are the mean (± SEM) of two or three independent experiments. The values between GUINEA-18 and GUINEA-18/MR7665’region were not statistically significant (n.s.), using the unpaired t test. (TIF) [file pntd.0012066.s008.tif]

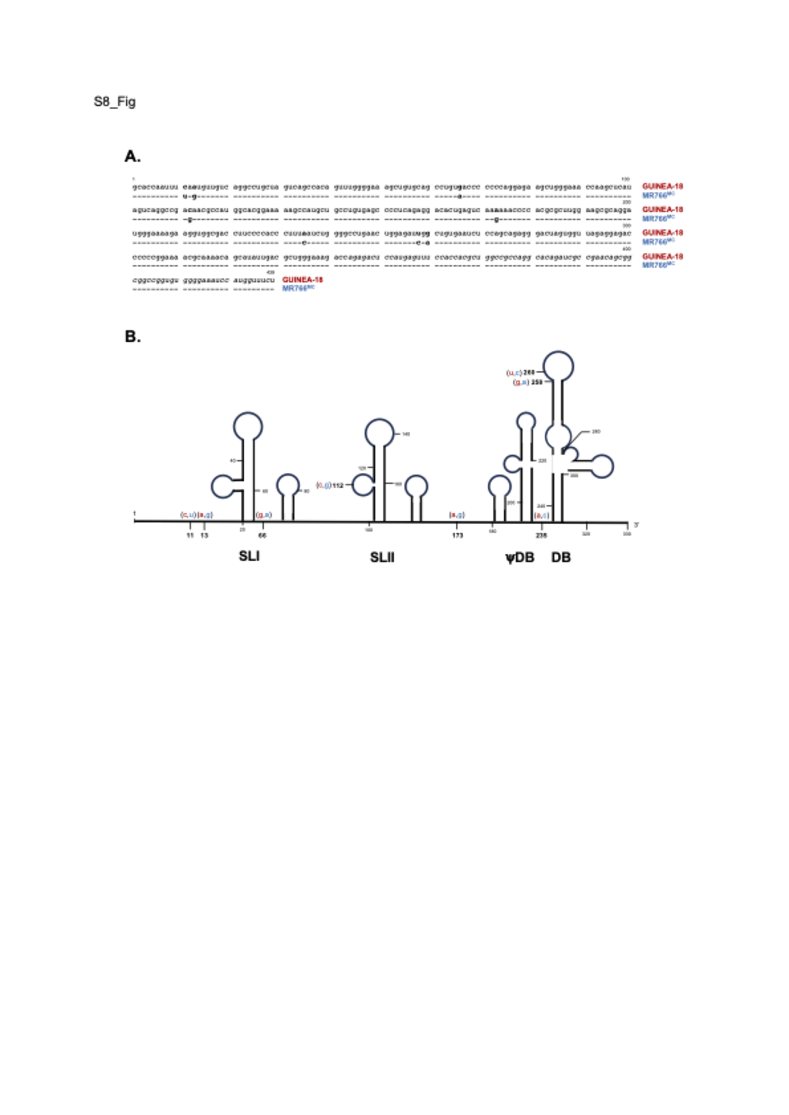

Supplement: S8 Fig — In (A), 3’NCR sequence alignment of GUINEA-18 and MR766MC. The lacking 3’ end of ZIKV-15555 3’NCR (Accession n° MN025403) was completed in GUINEA-18 by nucleotides 344 to 439 (in italic) from MR766MC corresponding to nucleotides 10722 to 10807 of MR766-NIID genomic RNA (Accession n° LC002520). Mutations between GUINEA-18 (red) and MR766 (blue) are indicated in bold. In (B), the positions of seven mutations that differentiate the first 330 nucleotides of 3’NCR from GUINEA-18 (red) and MR766 (blue). The positioning of mutations on the predicted stem-loops (SLI and SLII), dumbell (DB) and pseudo-DB (ψDB) structures is based on the predicted structure of ZIKV 3’NCR [33]. (TIF) [file pntd.0012066.s009.tif]

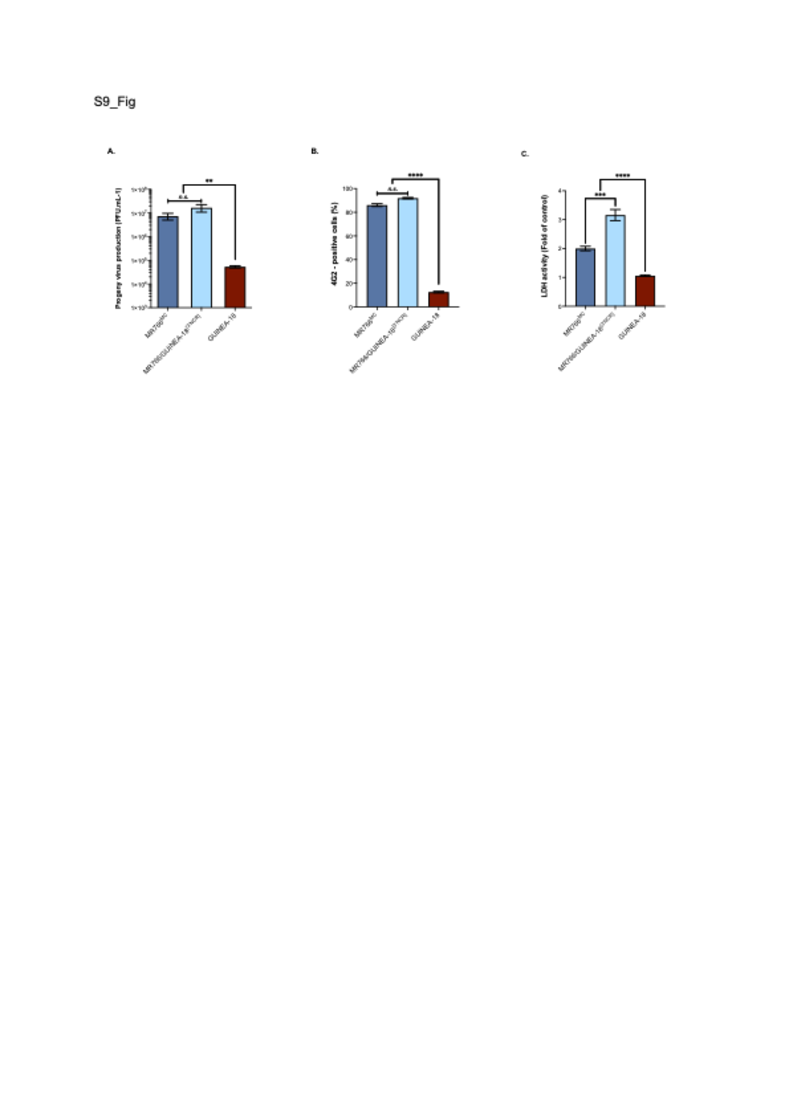

Supplement: S9 Fig — VeroE6 cells were infected for 48h with chimeric MR766/GUINEA-18[3’NCR] virus or parental viruses (MR766MC and GUINEA-18) at an m.o.i. of 0.1. In (A), virus progeny production. In (B), FACS analysis was performed with anti-E mAb 4G2. In (C), LDH activity was measured at 72h p.i. The results are the mean (± SEM) of two independent experiments. Asterisks indicate that the differences between experimental samples at each time point are statistically significant, using the unpaired t test and one-way ANOVA (**** p < 0.0001; *** p < 0.001; ** p < 0.01, n.s.: not significant). (TIF) [file pntd.0012066.s010.tif]

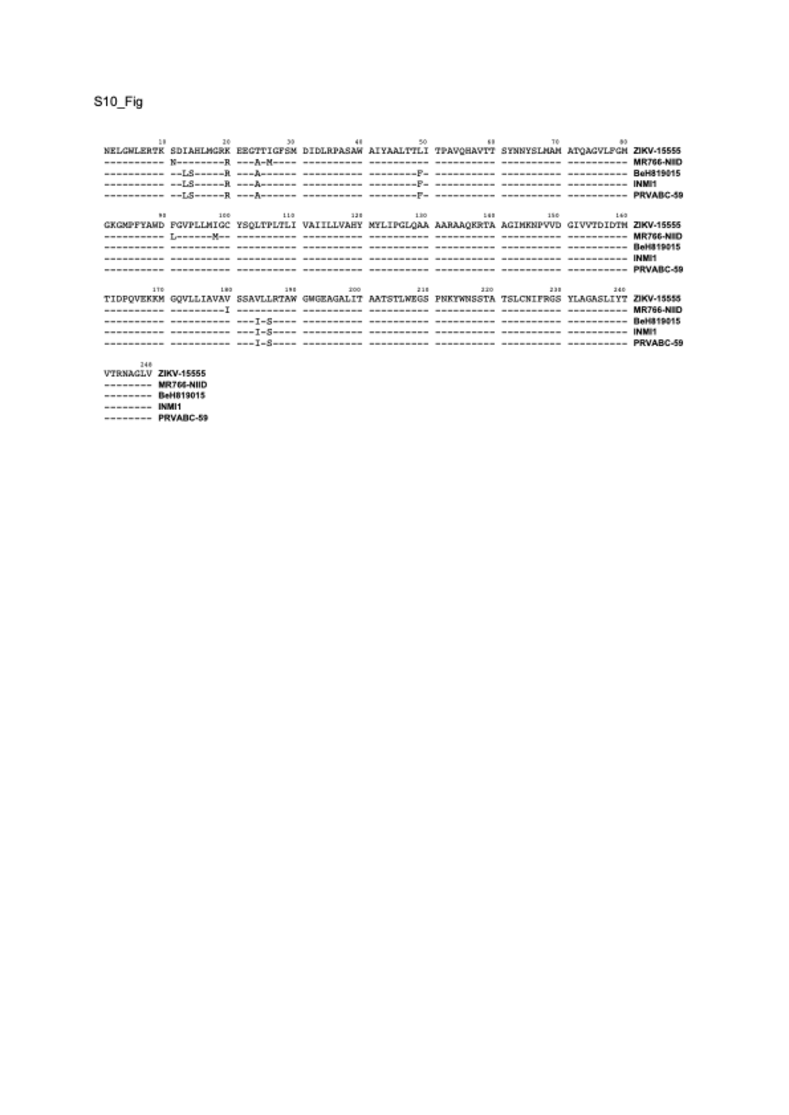

Supplement: S10 Fig — In (A), three-dimension structure prediction server Phyre2 was used to predict the 3D structures of the N-terminal region followed by the transmembrane helix (TM1) of ZIKV-15555 NS4B protein. The 3D viewing of the predicted structure was performed using the JSmol molecular visualization system. The clusters of residues GWLETRTKSDIAHLM (NS4B-3/17) and PASAWAIYAALTTLI (NS4B-36/50) have propensity for forming α-helical structure (helix α1) and TM1, respectively. The central disordered structure corresponding to the cluster of GRKEEGTTIGFSMDIDLRP (NS4B-18/35) residues includes the three mutations at positions 20/24/26 that differentiate ZIKV-15555 from MR766. In (B), sequence alignment of NS1 proteins from African ZIKV strains ZIKV-15555 and MR766-NIID, and epidemic Asia/America ZIKV strains BeH819015 (Accession n°KU365778), INTMI1 (Accession n°KU991811), and PRVABC-59 (Accession n°KU591215). (TIF) [file pntd.0012066.s011.tif]

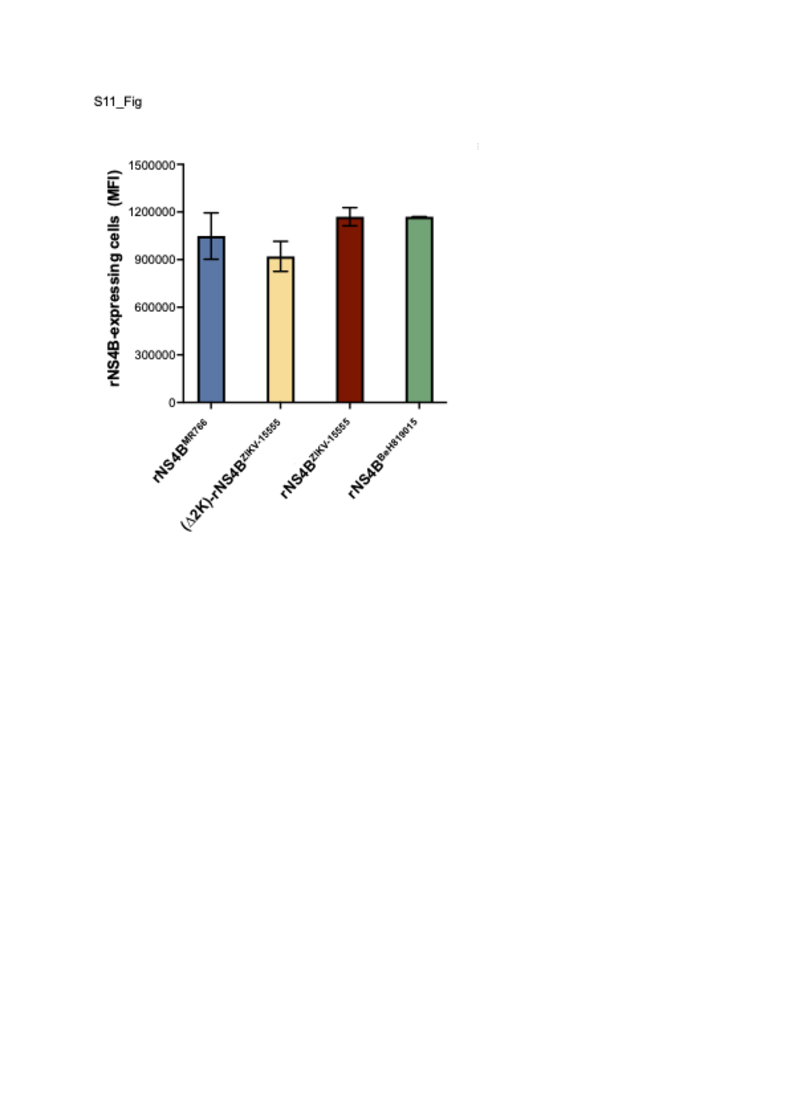

Supplement: S11 Fig — A549 cells were transfected for 24h with plasmids expressing recombinant 2KNS4B (rNS4B) proteins from West African ZIKV strains MR776 and ZIKV-15555, and epidemic Asian/American ZIKV strain BeH819015. A plasmid expressing ZIKV-15555 rNS1 protein without the N-terminal 2K peptide [(Δ2K)-rNS4BZIKV-15555] served as control. FACS analysis was performed using anti-FLAG antibody and the mean of fluorescence intensity (MFI) of transfected cells positive for FLAG-tagged rNS1 protein expression was measured as arbitrary units. (TIF) [file pntd.0012066.s012.tif]

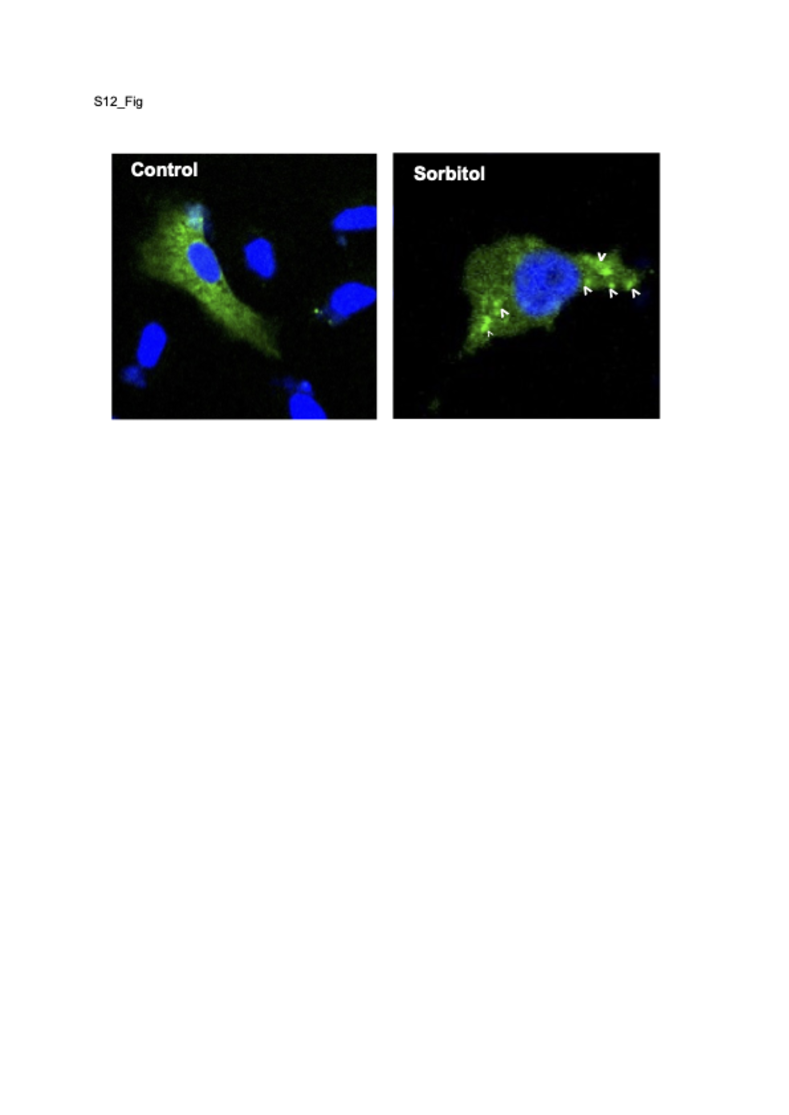

Supplement: S12 Fig — A549 cells were transfected for 24h with a plasmid expressing eGFP-G3BP fusion protein and then incubated with 0.4M sorbitol for 1.5h (sorbitol) or mock-treated (control). Nuclei were stained with DAPI (blue). The cells were processed for confocal microscopy analysis. The blank arrow heads indicate the eGFP-positive condensates. (TIF) [file pntd.0012066.s013.tif]
